# Pleiotropic Effects of Deubiquitinating Enzyme Ubp5 on Growth and Pathogenesis of *Cryptococcus neoformans*

**DOI:** 10.1371/journal.pone.0038326

**Published:** 2012-06-14

**Authors:** Wei Fang, Michael S. Price, Dena L. Toffaletti, Jennifer Tenor, Marisol Betancourt-Quiroz, Jennifer L. Price, Wei-hua Pan, Wan-qing Liao, John R. Perfect

**Affiliations:** 1 Shanghai Key Laboratory of Molecular Medical Mycology, PLA Key Laboratory of Mycosis, Institute of Dermatology and Mycosis of Changzheng Hospital, Second Military Medical University, Shanghai, China; 2 Department of Medicine, Duke University Medical Center, Durham, North Carolina, United States of America; University of Minnesota, United States of America

## Abstract

Ubiquitination is a reversible protein modification that influences various cellular processes in eukaryotic cells. Deubiquitinating enzymes remove ubiquitin, maintain ubiquitin homeostasis and regulate protein degradation via the ubiquitination pathway. *Cryptococcus neoformans* is an important basidiomycete pathogen that causes life-threatening meningoencephalitis primarily in the immunocompromised population. In order to understand the possible influence deubiquitinases have on growth and virulence of the model pathogenic yeast *Cryptococcus neoformans*, we generated deletion mutants of seven putative deubiquitinase genes. Compared to other deubiquitinating enzyme mutants, a *ubp5*Δ mutant exhibited severely attenuated virulence and many distinct phenotypes, including decreased capsule formation, hypomelanization, defective sporulation, and elevated sensitivity to several external stressors (such as high temperature, oxidative and nitrosative stresses, high salts, and antifungal agents). Ubp5 is likely the major deubiquitinating enzyme for stress responses in *C. neoformans,* which further delineates the evolutionary divergence of *Cryptococcus* from the model yeast *S. cerevisiae*, and provides an important paradigm for understanding the potential role of deubiquitination in virulence by other pathogenic fungi. Other putative deubiquitinase mutants (*doa4*Δ and *ubp13*Δ) share some phenotypes with the *ubp5*Δ mutant, illustrating functional overlap among deubiquitinating enzymes in *C. neoformans*. Therefore, deubiquitinating enzymes (especially Ubp5) are essential for the virulence composite of *C. neoformans* and provide an additional yeast survival and propagation advantage in the host.

## Introduction

*Cryptococcus neoformans* is a very important basidiomycete fungal pathogen, which can cause life-threatening meningoencephalitis primarily among immunocompromised hosts. It has been estimated that there are over 1 million new cases of cryptococcal infection in HIV-infected patients each year, and approximately 625,000 deaths are associated with cryptococcal meningoencephalitis [1]. Furthermore, cryptococcosis has also become one of most common invasive fungal infections in patients with solid organ transplantations or other immunosuppressive conditions [2]. Finally, an outbreak of cryptococcal disease among immunocompetent humans and animals on Vancouver Island and the Pacific Northwest, USA since 1999 caused by *Cryptococcus gattii* (a species that is closely related to *C. neoformans*) emphasizes that this infection can break out of the classic opportunistic risk groups [3]. Undoubtedly, cryptococcosis has become a major pathogenic burden on human health.

*Cryptococcus* can overcome various hostile conditions in vivo (such as high temperature, oxidative and nitrosative stresses, nutrient starvation, hypoxia, and high CO_2_) to produce disease [4], [5]. Several pathogenic factors contribute to the rapid adaptation of *Cryptococcus neoformans* inside the mammalian host and have been genetically characterized, including its polysaccharide capsule [6], melanin [7], ability to grow at 37°C [8], urease [9], and phospholipase [10] . In addition, substantial progress has been made in understanding the signal transduction pathways regulating virulence and sexual reproduction over the last decade [11], [12], [13]. Due to its well-defined sexual cycle, robust animal models and molecular biology tools, *C. neoformans* has become an excellent model for the study of fungal pathogenesis [14], [15]. However, these advances have confirmed the fact that fungal virulence is a very complicated phenotype. Identification of the essential complex regulatory mechanisms in control of the cryptococcal virulence composite and its networks will be helpful in identifying targets to abrogate disease caused by *C. neoformans*.

Ubiquitination is an essential posttranslational modification of cellular proteins in eukaryotic cells, which marks certain proteins for degradation by the proteasome and thus is a major regulator of protein activity and/or localization. Besides ubiquitin and the proteasome, the ubiquitin pathway also includes multiple enzymes such as E1 (ubiquitin activating enzyme), E2 (ubiquitin conjugating enzyme), E3 (ubiquitin ligase), and the DUBs (deubiquitinating enzymes or deubiquitinase) [16], [17]. Ubiquitination comprises a series of reactions, in which ubiquitin is activated first by E1, transferred to E2, and finally recognizes and tags specific substrate proteins for degradation via the proteasome with the help of E3. DUBs not only process the ubiquitin precursors into mature monomers, but also help to re-utilize ubiquitin by cleaving it from the substrate before targeted protein degradation. The ubiquitin-proteasome system plays a central role in a variety of cellular processes such as cell cycle progression, signal transduction, transcriptional control, DNA repair, and stress responses [17], [18].

DUBs belong to a highly-conserved protease superfamily that can be divided into five subclasses based on their catalytic domain structure: Ubiquitin C-Terminal Hydrolases (UCHs), ubiquitin-specific proteases (USPs), ovarian tumor proteases (OTUs), JAB1/MPN/Mov34 metalloenzymes (JAMMs) and Machado-Joseph disease proteases [19], [20]. A large number of DUB genes have been discovered in eukaryotes (including approximately 19 in *S. cerevisiae*, 22 in *Schizosaccharomyces pombe*, and 95 in the human genome), thus demonstrating their essential functions [20], [21]. In addition to maintaining ubiquitin homeostasis, DUBs also play important roles in proofreading some specific ubiquitinated substrates and rescuing them from proteolysis or the non-degradative pathway [19].

In the last few years, significant progress has been made in understanding the structures and biological functions of DUBs in *S. cerevisiae*, *S. pombe* and humans [22], [23], [21]. In *S. cerevisiae*, DUBs participate in regulating energy metabolism, sexual reproduction, as well as various stress responses [24], [25], [26], [27], [28]. For example, Doa4 and Ubp10 regulate the utilization of carbon and nitrogen sources, respectively [24], [27]. Deletion of *UBP14* leads to decreased meiotic efficiency, and a *doa4*Δ mutant shows a sporulation defect [25]. Most DUBs in *S. cerevisiae* are generally involved with the regulation of different stress responses [26], [29], [28]. It is noteworthy that the DUB-encoding gene *UBP15* not only regulates the stress response due to temperature changes and chemical stresses, but also plays important roles in the growth rate and cell size of *S. cerevisiae* [29], [30], [31]. Recently, studies have suggested that DUBs take part in pathogenesis of *C. neoformans* via regulating various stress responses and melanization [32], [33]. However, the networks and mechanisms of deubiquitinating enzymes as major controllers of the fungal virulence composite remain to be further understood.

In this study, we use *C. neoformans* as a model to investigate the role of the deubiquitinating enzyme family on fungal pathogenesis. We found that deletion of the putative DUB-encoding gene *UBP5* resulted in severely attenuated virulence and many distinct phenotypes, encompassing not only the defects in the classical pathogenicity factors such as capsule production and melanin synthesis, but also elevated sensitivity to several other important biological stresses and showed slower growth. In addition, we also confirmed that Ubp5 influences the sexual reproduction of this yeast. Our work illustrates the central importance of *UBP5* in adapting to hostile growth environments including infection sites in the host, and highlights the divergence of this yeast from other eukaryotes in the evolution of the DUB enzymes.

## Results

### Identification of DUB Homologues in *C. neoformans*

In order to identify genes important for maintenance of infection and survival in the central nervous system (CNS), we performed comparative transcriptional profiling experiments using WT strain H99 and previously described mutant strains during growth in ex vivo cerebrospinal fluid (CSF) and serum, and during human or rabbit infection. As shown in Table S2, 17 genes putatively involved in the ubiquitin-proteasome system were identified as being differentially expressed in these conditions. Therefore, to appreciate the function of this in vivo regulated ubiquitination system, we focused on the importance of deubiquitinating enzymes (DUB) in *C. neoformans* disease.

We identified 19 putative DUB proteins in the *C. neoformans* strain H99 genome belonging to four of five DUB subfamilies (UCH, USP, OTU, and JAMM) by homology to known DUBs from *S. cerevisiae* and *S. pombe*. Of these, 12 have been previously described via signature-tag mutagenesis and their roles in *Cryptococcus* pathogenesis have been ascertained [32]. We deleted two of these putative virulence-related DUB genes, *DOA4* and *UBP14*, in the WT strain H99 for use as controls in this study. Five of the remaining seven uncharacterized putative DUBs were deleted in the current study: Ubp5 (CNAG_05650), Ubp7 (CANG_03872), Ubp13 (CANG_01377), Otu1 (CANG_06428) and Rpn11 (CANG_07028). Of the two remaining genes (*UCH2*, CNAG_00989; *UBP10*, CNAG_01614), one (*UCH2*) may be an essential gene as we are unable to obtain a null mutant strain after numerous attempts. In contrast, putative null mutants of *UBP10* exhibit unusual cell morphology and have not been further characterized.

**Figure 1:**
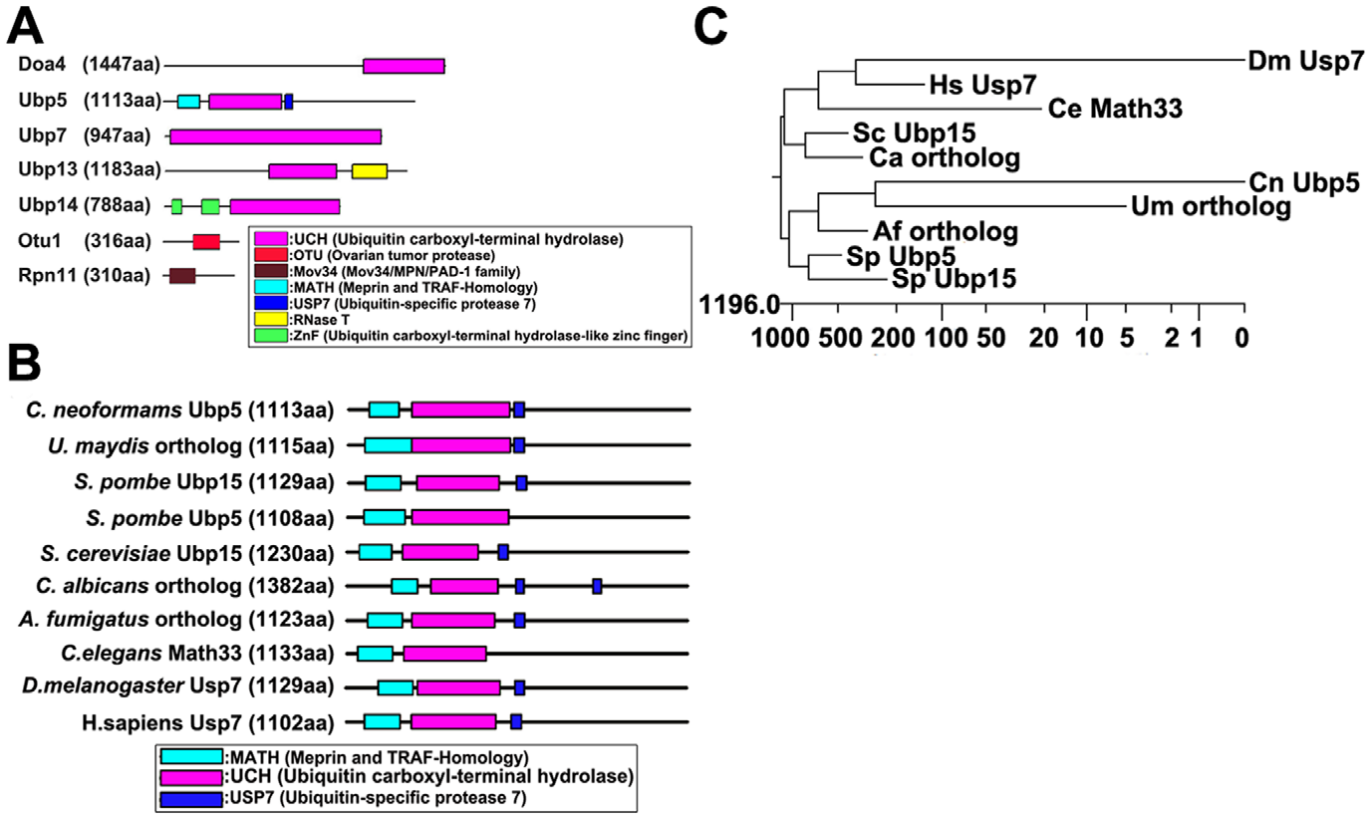
Structure analysis of deubiquitinating enzymes. A. Domain structure of cryptococcal deubiquitinating enzymes. 7 DUB enzymes in this study belong to three subfamilies (USP, OTU, and JAMM) on the basis of their catalytic domains. USP and OTU domain DUBs are cysteine proteases, JAMM domain DUB is metalloprotease. We retrieved domain architectures for each DUB using the Pfam databases (http://pfam.sanger.ac.uk/). B. Comparison of Ubp5 orthologs between *C. neoformans* and other species. Each Ubp5 ortholog diagram shows functional protein domains, which were identified by the Pfam database. C. Phylogenetic tree analysis of Ubp5 orthologs is depicted by Clustal W alignment from DNASTAR software (versions 6.13). Protein sequences of Ubp5 orthologs were retrieved from the following database: *C. neoformans* Ubp5 (CNAG_05650) from the *C. neoformans* var. grubii H99 database of the Broad Institute, and its orthologs among other eukaryotic species from the protein databank of NCBI website. Cn, *Cryptococcus neoformans*; Um, *Ustilago maydis*; Sp, *Schizosaccharomyces pombe*; Sc, *Saccharomyces cerevisiae*; Ca, *Candidia albicans*; Af, *Aspergillus fumigatus*; Ce, *Caenorhabditis elegans*; Dm, *Drosophila melanogaster*; Hs, *Homo sapiens*.

Bioinformatic analysis of the putative cryptococcal DUB proteins in this study showed that five of them belong to the USP subfamily (the largest one in *C. neoformans* includes 15 DUBs), while the other two belong to either the OTU or JAMM subfamilies. Many of these DUBs have amino-terminal extensions while some have carboxyl-terminal extensions (Figure 1A). Even within the same subfamily, their catalytic domains exhibit significant differences in length, suggesting that there must be different insertions in the catalytic domains of many isoforms. Previous studies have confirmed that DUBs do display specificity for both substrates and particular ubiquitin chain types, yet they all share the basic role of cleaving off ubiquitin from other adduct or fusion proteins [34], [20], [21]. These extensions and insertions may contribute to the functional diversity of the DUB enzymes. Interestingly, we found that deletion of *UBP5* generated many intriguing phenotypic changes in *C. neoformans* that were linked to virulence. Therefore, we focused in detail on *UBP5* for its relationship to pathogenesis.

We compared protein sequences of CNAG_05650 and Ubp5 orthologs from different eukaryotic species. Sequence analysis indicated that CNAG_05650 encodes 1113 amino acids in multiple domains, including an N-terminal MATH domain (the Meprin And TRAF-Homology domain, E-value: 3.4e-06), a UCH domain (Ubiquitin carboxyl-terminal hydrolase, E-value: 3.8e-59), and a C-terminal USP7 domain (Ubiquitin-specific protease 7, E-value:2.4e-12) (Figure 1A). Domain arrangement was similar to *S. cerevisiae* deubiquitinating enzyme Ubp15 with 38%, 54% and 44% identity in the three domains. An alignment of *C. neoformans* CNAG_05650 with orthologs from other eukaryotic species revealed conservation in protein domain structure (Figure 1B & 1C).

### Characterization of DUB Roles in Stress Responses

To investigate the roles of deubiquitinating enzymes in growth and virulence of *C. neoformans*, seven DUB genes were deleted in the serotype A strain H99 (Table 1). Targeted disruption was confirmed by PCR and Southern hybridization. Subsequently, we reconstituted the *ubp5*Δ mutant to WT via ectopic integration of the WT gene, and all the phenotypes shown for the *ubp5*Δ mutant were complemented in the *ubp5*Δ*+UBP5* reconstituted strain.

**Table 1:** Strains and plasmids used in this study.

Strain and Plasmid	Genotype	Reference
*C. neoformans*		
H99	*MATα*	[98]
KN99a	*MATa*	[101]
DF001	*MATαubp5::NEO*	This study
DF002	*MATa ubp5::NAT*	This study
DF003	*MATαubp5::NEO+ UBP5::NAT*	This study
DF004	*MATαdoa4::NAT*	This study
DF005	*MATαubp7::NEO*	This study
DF006	*MATαubp13::NEO*	This study
DF007	*MATa ubp14::NAT*	This study
DF008	*MATαotu1::NEO*	This study
DF009	*MATαrpn11::NEO*	This study
DF010	*MATa::NAT*	This study
TOC4	*MATαrim101::nat RIM101::NEO*	[88]
*ure1*Δ	*MATαure1::ADE2*	[9]
*cap59*Δ	*MATαcap59::HYG*	[101]
Plasmid		
pJAF1	NEO resistance gene	[88]
pCH233	NAT resistance gene	[88]
pUBP5-NAT	Modified pCH233 containing gene UBP5	This study

**Figure 2:**
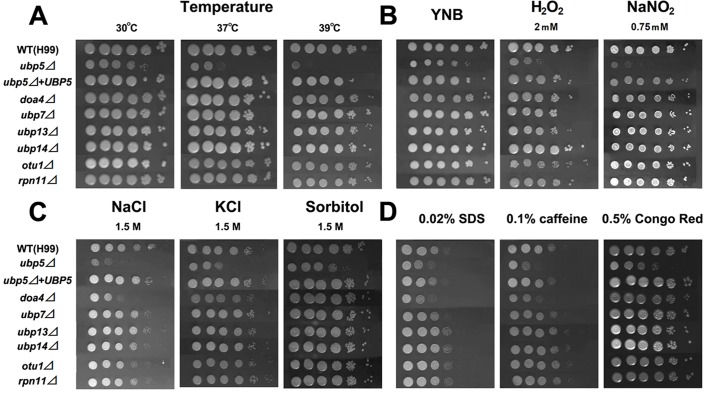
*UBP5* is involved in various stress responses of *C. neoformans*. A. Temperature sensitivity at 30°C, 37°C,39°C. B. Oxidative and nitrosative stress assay. C. High salts and osmotic stress assay. D. Cell-wall and cell-membrane stress assay. Strains were grown to saturation at 30°C in liquid YPD medium, 10-fold serially diluted (1–10^6^ dilutions), and spotted 3 µl cells on YPD or YNB agar (containing different stress-inducing agents). Incubated them for five days and then photographed.

Since the ubiquitin-proteasome system plays important roles in regulating stress responses in eukaryotes [33], we tested the sensitivity of each mutant to a variety of in vitro stresses. Unlike the WT and other DUB mutant strains, only the *ubp5*Δ mutant exhibited temperature sensitivity (ts), with a partial growth defect at 37°C and complete growth inhibition at 39°C (Figure 2A). The ts phenotype was not rescued by the addition of 1 M sorbitol (data not shown) as was observed for a *C. neoformans tps1*Δ mutant that is also thermosensitive [35]. Moreover, deletion of *UBP5* resulted in slower growth compared to WT even in rich medium at 30°C (Figure 2A and Figure S1). To examine whether this poor growth phenotype was due to carbon source preference, we also compared its growth using different carbon sources (YP agar medium with 3% galactose, 3% lactate, 3% glycerol, and 2% raffinose). No growth improvement was observed when the *ubp5*Δ strain grew in the media with alternate carbon sources (data not shown).

The *ubp5*Δ mutant also showed increased sensitivity to H_2_O_2_ (2 mM) and NaNO_2_ (0.75 mM), whereas the other DUB mutants exhibited no differences in sensitivity compared to the WT strain (Figure 2B). Likewise, the *ubp5*Δ mutant showed increased sensitivity to 1.5 M NaCl and 1.5 M KCl compared to WT, whereas the *doa4*Δ mutant exhibited increased sensitivity to elevated NaCl concentrations only (Figure 2C). Additionally, the *ubp5*Δ mutant showed increased sensitivity to 0.02% SDS, 0.1% caffeine or 0.5% congo red, whereas the *doa4*Δ mutant displayed increased sensitivity to 0.02% SDS and 0.1% caffeine (Figure 2D).

To further appreciate the impact of DUB enzymes on specific stress responses, all the mutants were exposed to various antifungal drugs: amphotericin B, caspofungin, fluconazole, or flucytosine. In vitro susceptibility testing was determined at 30°C according to the Clinical and Laboratory Standards Institute broth microdilution reference method (NCCLS, 2002). We observed that only the *ubp5*Δ mutant was hypersusceptible to all four antifungal agents compared with the WT strain, exhibiting an 8-fold reduction in MIC for flucytosine, 4-fold reduction in MICs for both fluconazole and caspofungin, and 2-fold reduction in MIC for amphotericin B (Table 2). The *ubp5*Δ+*UBP5* reconstituted strain completely restored WT sensitivity to the four antifungal drugs. Among the other DUB mutants, both the *doa4*Δ and *ubp13*Δ mutants showed increased susceptibility to fluconazole and caspofungin, but not to amphotericin B or flucytosine.

**Table 2:** In vitro susceptibility of mutant strains to four antifungal agents.

	MIC_80_(µg ml^−1^)			
Antifungal compounds	Amphotericin B	Fluconazole	Flucytosine	Caspofungin
H99	0.5	8	16	32
*ubp5*Δ	**0.25**	**2**	**2**	**8**
*ubp5*Δ*+UBP5*	0.5	8	16	32
*doa4*Δ	0.5	**4**	16	**16**
*ubp7*Δ	0.5	8	16	32
*ubp13*Δ	0.5	**4**	16	**16**
*ubp14*Δ	0.5	8	16	32
*otu1*Δ	0.5	8	16	32
*rpn11*Δ	0.5	8	16	32
ATCC22019	0.5	2	0.125	0.5

The MICs of four antifungal agents against *C. neoformans* WT, DUB mutants and reconstituted strains were determined by the Clinical and Laboratory Standards Institute broth microdilution refercence method (NCCLS, 2002), and *Candida parapsilosis* strain (ATCC22019) was used as a quality control strain.

Following exposure to external stressors, misfolded or damaged proteins trigger activation of the ubiquitin-dependent proteolytic pathway. Maintenance of a sufficient ubiquitin pool is essential for this process, and ubiquitin homeostasis is at least partly maintained by the activity of DUB enzymes [19]. To understand the relationship between *UBP5* deletion and ubiquitin stasis, we performed RT-PCR to examine the expression of *UBI4* (encoding polyubiquitin) in both *ubp5*Δ mutant and WT strains. *UBI4* expression was approximately 16-fold higher in *ubp5*Δ than in WT or reconstituted cells (Figure S3). Furthermore, deletion of *UBP5* led to greatly elevated *UBI4* expression at 37°C that peaked after 1 hr and then gradually declined. WT cells showed a gradual increase in *UBI4* expression at 37°C but its peak value was still significantly lower than that in *ubp5*Δ cells, suggesting that *UBP5* deletion may contribute to an increasing demand for polyubiquitin in *C. neoformans* cells to cope with high temperature and/or other stresses.

Taken together, deubiquitinating enzymes (especially Ubp5) appear to play important roles in regulating various stress responses. Deletion of Ubp5 made cryptococcal cells more susceptible to nearly all in vitro stresses that are closely related to pathogenesis compared to other DUB mutants. Therefore, we predicted that Ubp5 would play important roles in regulating pathogenesis of *C. neoformans.* Interestingly, phenotypes of several DUB mutants under stress conditions demonstrated both functional overlap and divergence within the deubiquitinating enzyme family for these pathogenic responses.

### Roles of DUBs in Capsule, Melanin and Urease Production in *C. neoformans*

**Figure 3:**
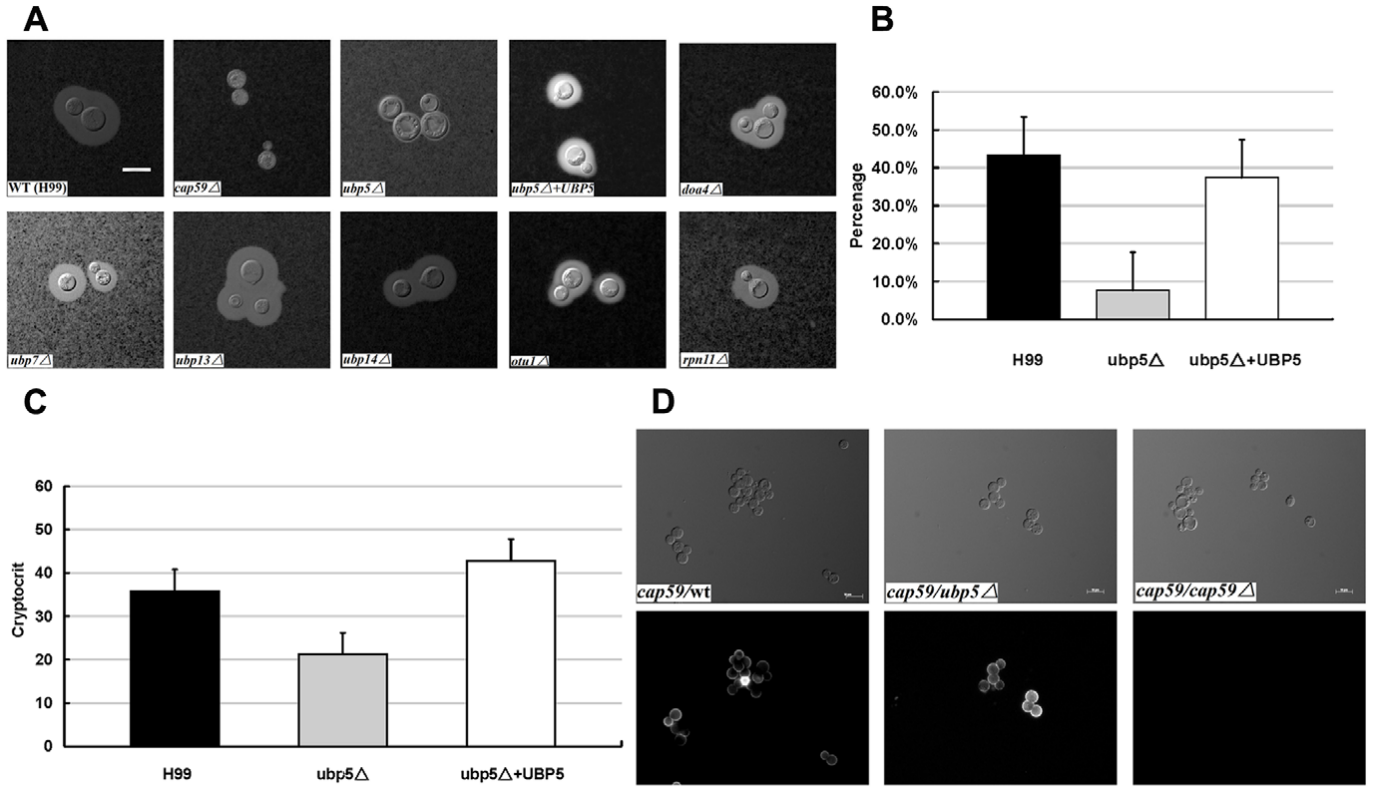
Disruption of *UBP5* significantly down-regulates the capsule production. A. *ubp5*Δ *mutants* have a capsule defect. The WT strain H99 and all the DUB mutants were cultured on DME medium for capsule production at 37°C for 3 days. Capsule was assessed by staining with India ink and visualizing at 100 magnification (scale bar  =  10 µm). B. Relative capsule volume detection. Total (cell and capsule) and cell-only diameter were measured via Photoshop Software for 50 cells for each strain. Then calculated the relative ratio of capsule with the formula ([Total Volume-Packed Volume]/Total Volume). After statistical analysis, the capsule production of *ubp5*Δ strain (7.7±0.48%) significantly decreased compared with either WT (43.4±6.41%) or reconstituted strain (37.4±8.01%) (*P*<0.001). C. Cryptocrit for Capsule Assay. After overnight growth, each culture was inoculated into tissue culture flasks with 20 mL DMEM medium in capsule-inducing condition for three days. Cells were killed with 10% formalin (V/V) for 5 minutes, and then counted. The samples were standardized to 3.2×10^8^ cells/mL. The difference between *ubp5*Δ (21.28±3.00) and complemented (42.8±0.84) or WT (35.8±1.48) strains was *P*<0.001. D. Capsule transfer assay. Capsule material secreted from wild-type or *ubp5*Δ cells into conditioned medium (CM) can be attached to acapsular acceptor cells (*cap59*Δ). *cap59*Δ cell cannot shed capsule polysaccharide, thus its CM was used as a negative control. Cells were labeled with monoclonal antibody mAb18B7 and Alexa Fluror anti-mouse IgG (secondary antibody) was used for immunofluorescence.

Several pathogenic factors are essential for *C. neoformans* to invade the host, such as capsule, melanin, and urease. When incubated under capsule-inducing conditions for 72 hrs, an obvious capsule defect was observed for the *ubp5*Δ mutant, whereas the reconstituted strain and other DUB mutants all showed WT capsule production (Figure 3A). In order to exclude the confounding effect of thermosensitivity, the *ubp5*Δ mutant was also examined in capsule-inducing medium at 30°C. Similarly, about 90% of *ubp5*Δ mutant cells displayed a significantly reduced capsule while 10% produced capsule similar to WT (data not shown). We measured the relative capsule volume (capsule diameter/total diameter) for the WT, *ubp5*Δ and *ubp5*Δ*+UBP5* strains, and found that the volume of the *ubp5*Δ capsule was significantly smaller than WT (*P*<0.001) (Figure 3B). These data were confirmed using an independent measurement of capsule volume based on packed-cell volume (Figure 3C **)**.

Since *C. neoformans* capsule is excreted from the cell via exocytosis for attachment to the cell wall [36], a small proportion of *ubp5*Δ mutant cells with similar capsule like WT suggested that Ubp5 might not actually regulate capsule synthesis, but instead regulate attachment. Therefore, a capsule transfer assay was also performed [37]. Conditioned medium (CM) was prepared as a source of capsule polysaccharide by growing WT and *ubp5*Δ mutant cells in DMEM medium for one week; CM from acapsular cells (*cap59*Δ) was used as a negative control. Indirect immunofluorescence showed that acapsular cells could attach the capsular polysaccharide from the *ubp5*Δ CM medium (Figure 3D). Therefore, the *ubp5*Δ mutant synthesized capsule but was defective in extracellular assembly of the capsule on the cell surface.

**Figure 4:**
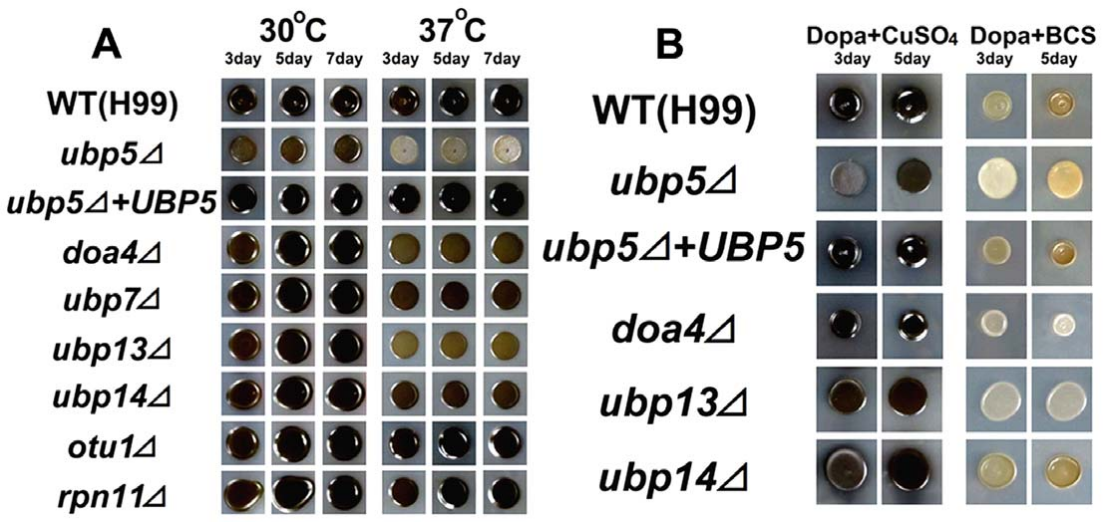
Melanin production assay of DUB mutants in *C. neoformans*. A. Melanin production assay. Strains were grown on L-DOPA medium alone at 30°C and 37°C. B. Regulation of melanization by copper availability. Mutants strains with hypomelanization were incubated onto DOPA agar containing + 10 mM CuSO_4_, or containing + 1 mM BCS, a copper chelator at 37°C.

Melanin is another classic virulence factor in *C. neoformans* [38]. We assessed melanization for each of the DUB mutants on DOPA medium at both 30°C and 37°C. The *ubp5*Δ mutant strain exhibited hypomelanization on melanin-inducing medium at 30°C, and this defect was exacerbated at 37°C (Figure 4). However, hypomelanizaion in three other DUB mutants (*doa4*Δ, *ubp13*Δ and *ubp14*Δ) occurred at 37°C, suggesting that these deubiquitinating enzymes also influence melanization at mammalian host temperature. Copper availability is a major factor influencing melanization in *C. neoformans.* Loss of several genes related to copper ion metabolism (such as *CCC2*, *ATX1* and *CUF1*) result in melanin defects in cryptococcal cells [39], [40], and many studies in *Saccharomyces* suggest that copper homeostasis is influenced by the ubiquitin system [41], [42]. Therefore, we hypothesized that DUBs might regulate melanization through their interaction with copper metabolism. We analyzed the melanization defect of the four DUB mutants by addition of exogenous CuSO_4_ and by chelation of copper ions from the media using bathocuproinedisulphonic acid (BCS). When copper ions were added into DOPA medium, melanin production was restored in all four DUB mutants (Figure 4B). However, after removing copper from DOPA medium, the *doa4*Δ and *ubp13*Δ mutant strains displayed completely non-melanized colonies whereas the *ubp5*Δ and *ubp14*Δ strains exhibited merely decreased melanization. Therefore, Ubp5 and Ubp14 may influence melanization by other mechanisms in addition to their influence on copper ion metabolism.

Urease is another important virulence factor of *C. neoformans*, which promotes increased CNS invasion and non-protective immune responses during lung infection [43], [44]. When incubated under Christianson’s urea agar, all the DUB mutants displayed WT urease activity as measured by the medium color change from yellow to bright pink (Figure S2). Therefore, these DUB enzymes do not influence urease activity.

### Ubp5 is Required for Sexual Reproduction of *C. neoformans*

**Figure 5:**
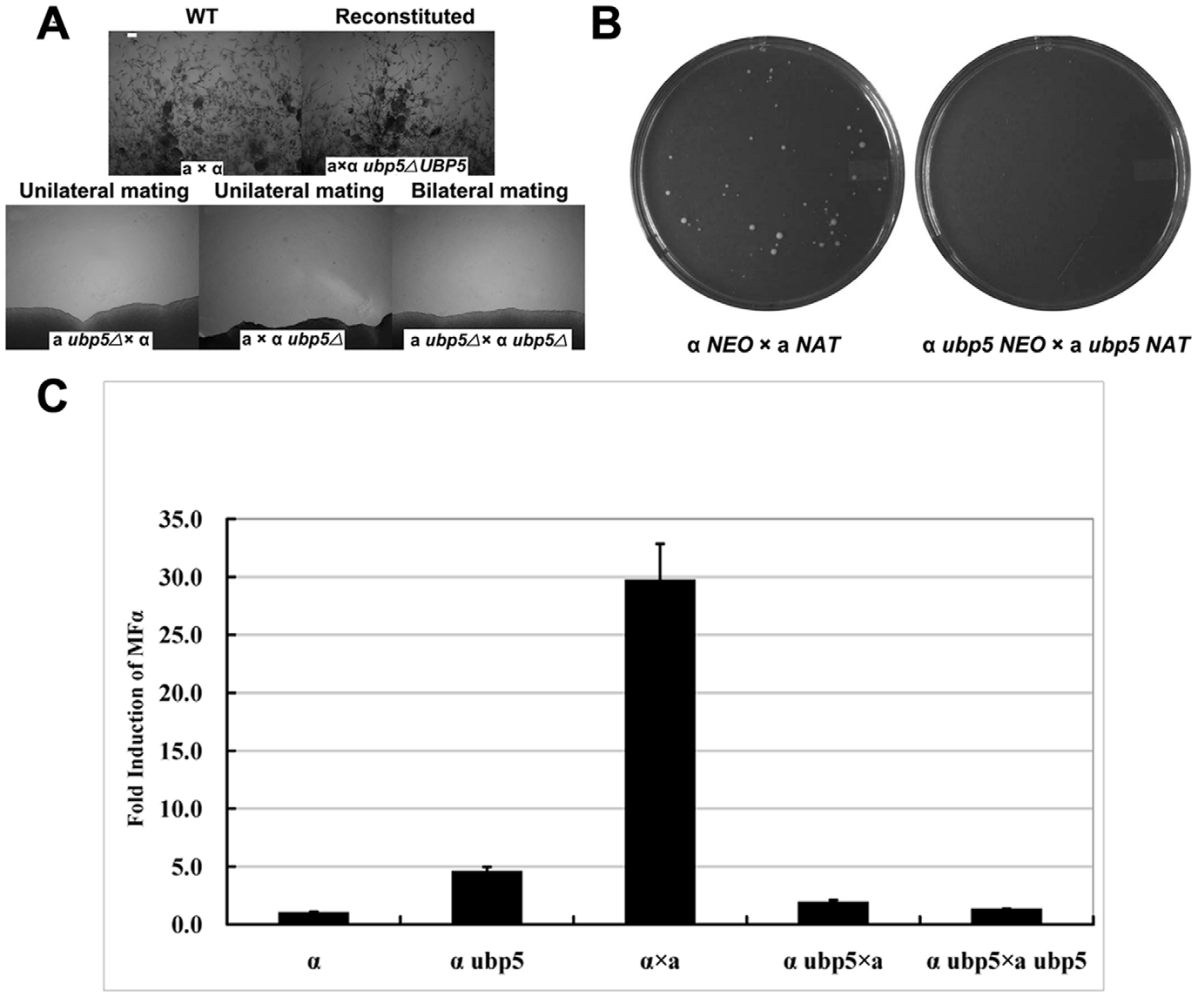
*C. neoformans* Ubp5 is required for mating. A. The *ubp5*Δ mutant is sterile in both unilateral and bilateral crosses. Reintroduction of the wild-type gene restores mating to a wild-type level. These fuzzy projections are mating-specific dikaryotic filaments. Mating assays were on V8 agar medium for 14 days at room temperature (scale bar  =  10 µm). B. The *ubp5*Δ mutant is defective in cell fusion. Wild type cells (H99 and KN99a) with different resistance genes (NEO and NAT) were set as a control. The *MAT*α and *MAT*a cells were mixed for 24 (WT strains) or 72 hours (*ubp*5Δ mutant strains), serial diluted, and plated onto YPD argar containing both nourseothricin and neomycin. Following fusion, dikaryotic cells containing both wild-type *MAT*α and *MAT*a cells were able to grow. However, no viable dikaryotic cells were on the double selective plate of *ubp*5Δ mutant. C. *MFα* pheromone gene expression was assessed in a WT and *ubp*5Δ mutant strains alone, and in unilateral and bilateral crosses. Cells were incubated on V8 mating medium for 24 h at room temperature. RNA was extracted, and *MFα* gene expression was detected by real-time PCR with primers MFα-a/b (Table S1).

The *UBP5* deletion was performed in both *C. neoformans* H99 (MATα) and KN99a (MATa) strain backgrounds. Sexual development and production of dikaryotic hyphae and basidiospores were examined in both unilateral crosses (*ubp5*Δ × WT) and bilateral crosses (MATa *ubp5*Δ × MATα *ubp5*Δ). Interestingly, no hyphae nor basidiospores were observed in unilateral crosses after two weeks co-incubation with mating partners (Figure 5A). In order to eliminate the effect of decreased growth rate on mating, we repeated the unilateral cross tests with a minor modification, mixing *ubp5*Δ and WT cells at 10∶1 ratio, and the result was still negative (data not shown) indicating that a single copy of *UBP5* is insufficient to allow mating in *C. neoformans*.

To identify where the defect in sexual reproduction occurred, we also performed a cell fusion assay. For this assay, strain DF001 (*MAT*α *ubp5*Δ NEO) was mated with either strain DF002 (*MAT*a *ubp5*Δ NAT) or DF010 (*MAT*a NAT) on V8 agar for 24 h or 72 h and then plated on double selective medium (YPD containing neomycin and nourseothricin). We observed that *ubp5*Δ strains did not form colonies in bilateral crosses, suggesting Ubp5 is absolutely required for cell fusion (Figure 5B). We also tested fusion in unilateral crosses between *ubp5*Δ and WT; lack of viable colonies from this mating indicates that *UBP5* in each mating partner is necessary for cell fusion (data not shown). This is consistent with *cpk1*Δ, *ste7*Δ, and *ste11*Δ mitogen-activated protein kinase (MAPK) pathway mutants, which also exhibit defects in fusion in unilateral crosses [45].

The *MFα* pheromone genes are important to initial cell fusion, and are induced by nutritional limitation and co-incubation with MATa cells [46]. *MFα* induction was assessed by real-time PCR in WTα and *ubp5*Δα mutant strains alone, and in mixed cell populations of unilateral and bilateral crosses. After 24 h co-culture with WT MATa cells, a dramatic induction of the *MFα* pheromone gene occurred in the WT MATα strain (Figure 5C) consistent with a previous report [46]. In contrast, when *ubp5*Δα mutant cells were co-incubated with WT or *ubp5*Δ mutant MATa cells, *MFα* expression showed no induction (Figure 5C). In addition, *MFα* transcription induced by limited nutrition alone was slightly higher in the *ubp5*Δ mutant than in WT. These results indicate that Ubp5 plays a complicated role in pheromone expression, which is not only required for induction of *MFα* transcription during mating but also negatively regulates its expression under nutrient-limited conditions.

### Roles of DUBs on Cryptococcal Survival in Macrophages

**Figure 6:**
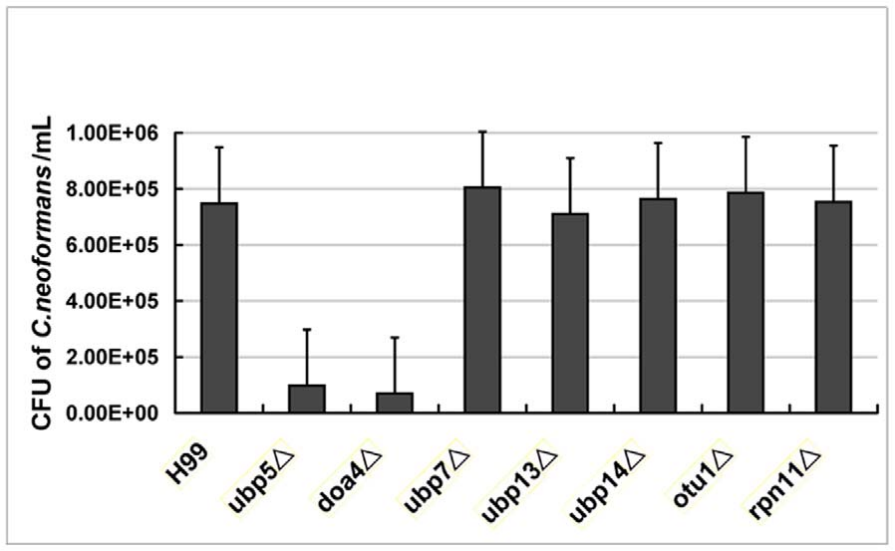
Ubp5- and Doa4-mediated survival in macrophages. Activated J774A.1 macrophages were co-incubated with the WT, all the DUB mutants and complemented strains of *C. neoformans* for 2 hours at 37°C in 5% CO_2_. Extracellular yeasts were then removed, and cocultures were incubated overnight under the same conditions. Following co-incubation, macrophages were lysed via 0.05% SDS and viable *C. neoformans* colonies were counted on YPD plates after 3–4 days’ growth at 30°C. Each strain was tested in triplicate (*P* ***_ubp5_***_Δ_ = 0.003**,** *P* ***_doa4_***_Δ_<0.001).

*C. neoformans* is a facultative pathogen, which can proliferate in both intracellular and extracellular environments [47]. Macrophages are primary professional phagocytes in the host, and also important for mediating the dissemination of cryptococcal cells from the lungs to the brain [48]. Therefore, we evaluated the DUB mutants for their ability to survive inside macrophages. The *ubp5*Δ and *doa4*Δ mutants exhibited significant reduction (87% and 92%, respectively) in their intracellular survival after co-inoculation with activated macrophages in DMEM for 24 hours, while other DUB mutants displayed WT survival (Figure 6). Interestingly, ectopic integration of *UBP5* failed to restore intracellular survival in macrophages in spite of restoring all in vitro phenotypes tested.

Multiple virulence factors of *C. neoformans* contribute to its intracellular replication inside macrophages, including production of capsule, melanin, and a variety of secreted enzymes [49], [47], [50], [51]. Obviously, lower intracellular survival of the *ubp5*Δ mutant might result from a complex of several virulence-related phenotypic changes such as capsule and melanin defects, hypersensitivity to nitric oxide or hydrogen peroxide stress, slower growth rate, as well as decreased thermotolerance. However, the *doa4*Δ mutant exhibited significantly impaired intracellular survival despite displaying WT phenotypes for many of these virulence-related traits. Therefore, we hypothesize that Doa4 may regulate intracellular proliferation of *C. neoformans* by as yet additional undefined means.

### Influence of Ubp5 on in vivo Virulence

**Figure 7:**
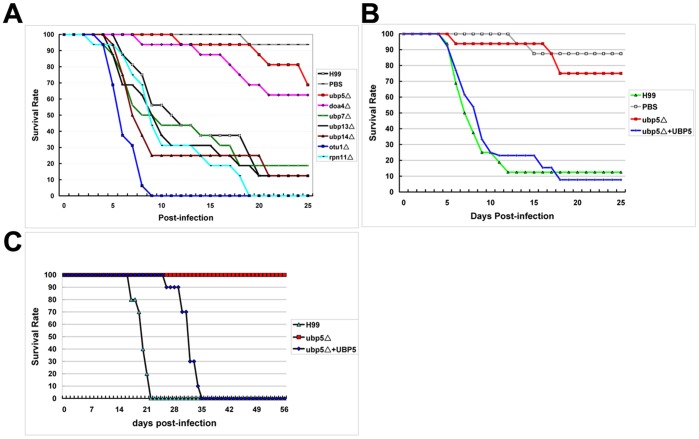
In vivo virulence assay of DUB mutants in *G. mellonella* and mouse infection models. A. Screening assay of DUB mutant strains in the larvae model revealed various degrees of virulence. B. Reconstituted strain (*ubp5*Δ*+UBP5*) completely restored the virulence in *G. mellonella* killing assay. C. Deletion of Ubp5 exhibited significantly attenuated virulence in murine inhalation infection model.

*Galleria mellonella* is a useful non-vertebrate model for high-throughput screening of in vivo virulence of *C. neoformans* [52], [53], [54]. In order to assess whether deubiquitinating enzyme deletions result in attenuated virulence, we employed a *G. mellonella* survival assay to assess our seven DUB enzyme mutant strains. Various degrees of virulence attenuation were observed in this invertebrate model (Figure 7A). The *ubp5*Δ and *doa4*Δ mutants showed a significant virulence attenuation: mean survival was 23.6±0.94 days for *ubp5*Δ (*P*<0.001) and 21.7±1.25 days for *doa4*Δ (*P* = 0.001) compared to 13.7±1.6 days for WT. In contrast, the *otu1*Δ mutant exhibited hypervirulence (6.4±0.36d, *P*<0.001) compared to WT strain (13.7±1.6d). None of the remaining mutants exhibited altered virulence compared to WT. These results are consistent with the macrophage killing assay, and both assays illustrated that different DUB enzymes impact discrete aspects of *C. neoformans* virulence despite their overarching functional similarities.

The ability to survive at mammalian body temperature is required for pathogenicity of *C. neoformans*; thermosensitive strains are attenuated in the mammalian host. Since deletion of *UBP5* impaired growth of *C. neoformans* at 37°C in vitro, we next assessed the virulence of the *ubp5*Δ mutant in a murine inhalation model of cryptococcosis. Female A/Jcr mice (10 per strain) were inoculated per nasally with 10^5^ CFU of the wild-type, *ubp5*Δ mutant, or *ubp5*Δ*+UBP5* reconstituted strains. Mice were monitored for survival and sacrificed at predetermined clinical endpoints. In contrast to the WT infected mice that exhibited complete mortality by 22 days post-inoculation, none of *ubp5*Δ-infected mice showed symptoms even after eight weeks post-infection, demonstrating a major virulence defect (*P*<0.001) (Figure 7C). Assessment of fungal burden in different organs showed that in comparison to WT or reconstituted-strain infected mice, viable cells were discovered from the lungs of mice infected by the *ubp5*Δ mutant but not the spleens or brains. Clearly, the *ubp5*Δ mutant is severely attenuated in the mammalian host for dissemination and disease even though the mutant exhibited prolonged survival in the lung. In contrast to the macrophage killing assay, reconstitution of *UBP5* into the *ubp5*Δ mutant partially restored its virulence in mice while completely restoring the *G. mellonella* virulence and other in vitro phenotypes (Figure 7B and C).

### *UBP5* Deletion Effects on the *C. neoformans* Transcriptome

To further gain insight into the molecular basis for the pleiotropic phenotypes caused by *UBP5* deletion, we carried out a microarray analysis of the differential genome-wide transcription profile of the *ubp5*△mutant versus its isogenic WT parental strain under both ambient and elevated temperature (37°C). These expression data have been submitted to the GEO database (http://www.ncbi.nlm.gov/gds) under accession number GSE35067. Among 10601 probes on the array, approximately 21% (1,463 genes) of the *C. neoformans* transcriptome showed significantly different expression patterns in the *ubp5*△mutant compared to the WT (*P*<0.05). Since the *ubp5*△mutant exhibited decreased melanin production at 37°C, the transcriptional change of the acid laccase-encoding gene CNM02420 (0.27-fold down-regulation, *P* = 5.82E-01) was selected as a reference cutoff. Approximately 653 genes were identified as biologically significant based on these criteria.

We specifically assessed how *ubp5* mutation affects gene transcription pattern in *C. neoformans* at 30°C. Genes regulated by DUB enzyme Ubp5 deletion covered a wide variety of functional categories (Table 3). First, a group of genes involved in nutritional metabolism exhibited differential expression in the *ubp5*△ mutant compared to WT. Several enzymes in carbon utilization (e.g. sorbitol, alcohol and fatty acid) and ion stasis (e.g. sodium, calcium and iron) were up-regulated while a number of nitrogen-metabolite transporters (e.g. ammonium, spermine and urea) were repressed, suggesting that *UBP5* deletion may alter metabolic flux in *C. neoformans*. Furthermore, several genes involved in stress defenses, such as *CNAG_07836* (NAD binding dehydrogenase), *CNL03740* (catalase), *SOD2* (manganese superoxide dismutase) [55], *AIF1* (apoptosis-inducing factor) [56] and *QDR3* (Multidrug transporter of the major facilitator superfamily), were differentially expressed by *UBP5* mutation. Ubp5 inactivation also resulted in many up-regulated genes associated with cell wall/membrane organization. This may partially explain the increased susceptibility to multiple cell-wall stressors and antifungal agents in the *ubp5*△ mutant (Figure 2 and Table 2). Finally, Ubp5 inactivation also led to differential expression of several genes involved in several basic cellular processes such as DNA replication, transcriptional regulation and cell cycle, and may explain the decreased growth rate at 30°C.

**Table 3:** Ubp5p-dependent gene expressions at 30°C.

Category	Gene ID*	Fold change ubp5?/WT	P value	Functional annotation*
Carbohydrate/amino acid metabolism	CNAG_00269	1.80	8.30E-09	Sorbitol dehydrogenase
	CNC05840	1.50	4.60E-07	Glycosyl hydrolase
	CNAG_06035	1.49	2.00E-08	Alcohol dehydrogenase
	CNC01140	1.38	3.00E-10	Lipase
	CNB00370	1.29	1.80E-07	Fatty acid beta-oxidation-related protein
	CNE01100	0.71	1.50E-09	long-chain-fatty-acid-CoA ligase
	CND00530	0.72	8.90E-07	Urea transporter
	CNC06440	0.70	4.80E-08	Inositol-3-phosphate synthase
	CNJ02210	0.63	7.10E-07	Spermine transporter
	CNJ02220	0.59	1.70E-09	Adenine nucleotide transporter, involved in beta-oxidationof medium-chain fatty acid
	CNJ01880	0.50	4.40E-09	Ammonium transporter (AMT2)
Cell wall/membrane organization	CNE03150	2.73	7.00E-12	1,3-beta-glucanase, involved in cell wall beta-glucanassembly (Exg1p)
	CNE03240	2.54	2.00E-18	Chitin synthase (Chs7p)
	CNAG_06031	1.82	2.00E-12	Beta-glucan synthesis-associated protein
	CNAG_04270	1.54	1.80E-09	SNAP receptor, mediate membrane fusion
	CNI02290	1.47	4.80E-08	Polysaccharide synthase (Cps1p)
	CNF01800	1.45	4.70E-07	Chitin deacetylase
	CNN00660	1.42	1.30E-08	Glucan 1,3 beta-glucosidase protein putative
Ion transport andmetabolism	CNL05450	2.30	8.00E-10	Sodium, inorganic phosphate symporter
	CNAG_04337	1.65	1.00E-14	Cation diffusion facilitator
	CNK01330	1.53	4.00E-11	Calcium ion transporter
	CND00150	1.52	5.40E-09	Ferric chelate reductase (Fre7p)
	CNE04050	1.43	2.00E-12	Iron ion transport-related protein
DNA replication and repair	CNAG_04696	1.91	1.00E-13	DNA clamp loader
	CNI04060	1.38	5.00E-10	Mitochondrial genome maintenance protein
	CNN01100	0.73	1.10E-06	Telomere maintenance protein
	CNN01650	0.62	4.70E-08	DNA dependent ATPase for DNA repair (Rad26p)
Transcriptional regulation	CNK03060	1.43	8.40E-09	C2H2 zinc finger protein Zas1
	CNAG_04403	1.43	9.80E-08	CCCH zinc finger protein
	CNB03490	0.64	3.70E-08	Transcriptional regulator
Stress response	CNAG_07836	1.94	2.00E-12	NAD binding dehydrogenase with oxidoreductase activity
	CNAG_04521	1.47	3.70E-09	Apoptosis-Inducing Factor (Aif1p)
	CNL03740	1.36	3.90E-07	Catalase
	CNI01590	1.29	1.00E-08	Manganese superoxide dismutase (SOD2p)
	CNL04910	0.66	1.10E-09	Multidrug transporter of the major facilitator superfamily (Qdr3p)
Signal pathway	CND06250	1.43	5.80E-06	MAP kinase kinase kinase
	CNAG_00802	1.39	3.90E-06	Calcineurin-binding protein (Cbp1p)
Cell cycle	CNG01990	1.62	8.00E-11	G1/s-specific cyclin pcl1 (cyclin hcs26)
	CNI01430	1.32	7.70E-06	Cyclin-dependent protein kinase regulator
Other	CNE02820	1.59	1.30E-09	AFG1-family ATPase
	CNAG_06009	1.53	1.10E-09	Cyclohydrolase
	CNC02450	1.48	1.50E-06	Water channel
	CNL04120	0.54	2.00E-10	Adenosylmethionine-dependent methyltransferase

* Gene ID and functional annotation obtained from NCBI database (http://www.ncbi.nlm.nih.gov/) or Broad database (http://www.broadinstitute.org/annotation/genome/cryptococcus_neoformans/) with additional hand editing.

We also examined gene expression at 37°C. Several novel findings were obtained from this analysis (Table 4). First, more genes involved in stress responses, such as *TPS2*, *CNB02790* (alpha, alpha-trehalase), *GST3* and *GST6* (glutathione S-transferase) were differentially expressed at 37°C compared to 30°C. Second, high temperature further inhibited some genes related to virulence factors such as *CAP64* (capsular-related protein) and *FET3* (acid laccase). In addition, a large number of genes involved in nucleotide metabolism, RNA processing and modification, ribosome synthesis and cell division exhibited significant reduction in *ubp5*△ at 37°C.

**Table 4:** Ubp5p-dependent gene expressions at 37°C.

Category	Gene ID*	Fold change ubp5?/WT	P value	Functional annotation
Carbohydrate/amino acidmetabolism	CNI00320	1.99	1.10E-07	Glyceraldehyde 3-phosphate dehydrogenase
	CNE03150	1.53	7.60E-06	Cellulase (167.m03461)
	CNA00470	1.52	1.10E-09	Fructose-bisphosphatase (181.m07830)
	CNAG_02225	1.41	1.60E-08	Cellulase
	CNF03760	1.40	2.30E-07	Carboxypeptidase C
	CNN00510	1.37	2.50E-08	L-fucose permease
	CNG01150	0.73	4.70E-06	Spermidine synthase
	CNAG_01118	0.72	5.40E-08	Amino acid transporter
	CNA08130	0.72	2.90E-08	2-hydroxyacid dehydrogenase
	CNL04780	0.64	1.90E-08	Carbonic anhydrase protein
	CNJ00690	0.59	1.20E-09	Uracil permease
Nucleotide metabolism	CNC04630	0.71	1.80E-06	Thymidylate kinase
	CNE00200	0.69	9.80E-08	Transferase purine nucleotide biosynthesis
	CNJ01230	0.64	1.30E-09	Thymidylate synthase
RNA processing and modification	CNF00090	0.70	7.00E-11	rRNA modification-related protein
	CNE01090	0.69	6.50E-09	rRNA primary transcript binding protein
	CNH02690	0.68	7.30E-08	ATP-dependent rRNA helicase (Spb4p)
	CNG03270	0.66	2.70E-07	RNA-3-phosphate cyclase rRNA processing
	CNJ00110	0.65	4.70E-08	rRNA (adenine-N6 ,N6-)-dimethyltransferase
Ribosome synthesis	CNC05320	0.66	1.00E-10	Mitochondrial 60 s ribosomal protein l38 (yml38)
	CNB01260	0.64	4.30E-09	Ribosomal large subunit biogenesis-related protein
	CNB05550	0.62	5.00E-11	Cytosolic large ribosomal subunit protein
Cell division and cell cycle	CNAG_06092	1.30	3.70E-07	Cyclin
	CNK02970	0.72	4.00E-06	Mitotic chromosome condensation-related protein
	CNAG_02267	0.72	2.00E-10	Spindle assembly checkpoint protein SLDB
	CNAG_00259	0.68	2.00E-10	Bud site selection protein
Cell wall/membrane organization	CNE01950	1.47	2.20E-08	UDP-N-acetylglucosamine transporter
	CNA06800	0.72	1.20E-07	CAP64 gene product (Cas31p)
	CNC06200	0.70	4.90E-09	Ergosterol biosynthesis-related protein
Transcriptional regulation	CNAG_04457	2.02	1.30E-07	Transcription factor activity
	CNAG_02877	1.60	2.40E-07	Sequence-specific DNA binding transcription factor activity
	CNC02990	0.63	8.10E-08	DNA-directed RNA polymerase
	CNAG_06916	0.60	1.80E-09	Zinc finger protein
Stress response	CNAG_03765	1.37	6.00E-09	Trehalose-phosphatase (Tps2p)
	CNA07730	1.34	2.30E-09	Multidrug resistance protein 1
	CNAG_01893	1.33	2.60E-07	Glutathione S-transferase 3 (Gst3p)
	CNB02790	1.30	5.60E-09	Alpha,alpha-trehalase
	CNM02420	0.73	5.82E-01	Acidic laccase (Fet3p/5p)
	CNF02230	0.57	2.00E-11	Glutathione S-transferase 6 (Gst6p)

* The table does not list those differential genes overlapped with Table 3.

## Discussion

Modification of proteins by ubiquitination is a critical regulatory mechanism for various cellular processes. Proteomic studies indicate that about 1/5?1/6 of all proteins (1,075) are modified by ubiquitination in *Saccharomyces* [57]. Deubiquinating enzymes (DUBs), which mediate the removal and processing of ubiquitin from ubiquitin precursors, ubiquitin-protein conjugates, and unanchored ubiquitin chains, play essential roles in protein degradation by either the proteasome or other ubiquitin-dependent processes for their help in recycling ubiquitin. A previous cryptococcal study described the phenotype of 12 DUB gene deletion mutants regarding the classic pathogenicity factors (capsule production, melanin and high temperature growth) and in vivo virulence in the CMO18 strain background [32]. On the basis of that study and our own in vivo transcriptional analyses that suggested importance of this pathway for the virulence composite, we performed a comprehensive functional analysis of the DUB family in the model pathogenic yeast *C. neoformans*. *C. neoformans* possesses 19 putative DUB genes among which most appear to belong to the USP subfamily. In this report, we disrupted five of the remaining deubiquitinase genes in the H99 strain background, along with the creation of two confirmed virulence-related mutant strains (*doa4*Δ and *ubp14*Δ) in same background for use as controls [32]. None of these genes were essential and most DUB mutants did not exhibit significant growth defects under standard growth conditions. However, deletion of *UBP5* led to severely altered phenotypes, which illustrates that this deubiquitinating enzyme plays a vital but specific role in regulating cell propagation, sexual reproduction, stress response and pathogenesis of *C. neoformans*.

The first distinct phenotype in the *ubp5*△mutant is its poor growth performance even in rich medium (Figure 2A) and alternative carbon sources do not improve its growth (data not shown). This observation may be partially contributing to the disordered nutritional utilization caused by Ubp5 deletion (Table 3). Some deubiquitinating enzymes in *Aspergillus nidulans* and *S. cerevisiae* also play roles in regulating their utilization of carbon resources [58], [27]. On the other hand, the growth suppression of *ubp5*△ mutant is associated with its decreased proliferation rate (Figure S1), which is similar to the null mutant of *UBP15* (the orthologue of *CnUBP5*) in *S. cerevisiae* [59]. Through physical interaction with APC/C (anaphase-promoting complex/cyclosome, an ubiquitin-ligation enzyme) activator Cdh1p, ScUbp15p can reverse the degradation of cell cycle proteins via ubiquitination and drives the transitions through the different stages of the cell cycle [60], which then governs the rate of cell proliferation in *S. cerevisiae* [61]. In *C. neoformans*, Ubp5 inactivation contributed to differential transcription of several genes involved in cell cycle regulation. Therefore we hypothesize that Ubp5 may regulate cell division in a similar fashion in *C. neoformans*. The *ubp5*△mutant may be dependent on such regulatory mechanisms to reduce non-essential energy consumption and to meet enhanced energy demand due to high temperature, since Ubp5 deletion may disturb normal energy mobilization and utilization in *C. neoformans* (Table 3).

In addition to asexual propagation, we found that Ubp5 also plays an essential role in sexual reproduction and possibly production of one of the primary infectious propagules (basidiospores) in human cryptococcosis [62]. Sexual development of cryptococcal cells involves a series of fundamental events such as plasmogamy, karyogamy, meiosis and sporulation [63]. Our tests showed that the mating of *ubp5*Δ mutant with WT fails to produce any filaments, and appears to be blocked at the point of cell fusion. This is partially attributed to blocked *MFα* pheromone gene induction during mating (Figure 5). A recent study illustrates that the ubiquitin ligation enzyme Fbp1 also regulates sporulation in *fbp1*Δ bilateral crosses of *C. neoformans* [64]. However, it remains to be elucidated whether it works in concert with other genes such as the DUBs. Several ubiquitin-related proteins, such as Ubi4 (polyubiquitin), Doa4, and Ubp14 (deubiquitinating enzymes) were confirmed to function in the coordination of sexual reproduction in *S. cerevisiae*, where deletions of these genes lead to a sporulation defect [65], [25]. The pathway may be dependent on the degradation of MAPK pathway components or the transcriptional repressor of mating-type specific genes mediated by ubiquitination [66], [67], [68]. The ubiquitin pathway may also regulate sporulation in a similar way in *C. neoformans*. However, no sporulation defects have been shown in the ScUbp15 mutant as have been observed for the *C. neoformans* orthologue CnUbp5. This illustrates the functional divergence and redistribution among the deubiquitinating enzymes from different fungal species.

*C. neoformans* is a globally distributed and free-living pathogenic fungus with tenacious vitality, which is illustrated by its adaptation to dramatically different environments during infection within a mammalian host. Among many in vivo stressors for this yeast, increased environmental temperature is a major challenge that *C. neoformans* encounters upon initial entry into the host. We demonstrated that disruption of the *C. neoformans UBP5* gene confered a conditional temperature-sensitive growth defect. Reconstitution of the wild-type *UBP5* allele fully restored the ability of the *ubp5*Δ mutant to grow at 37°C. Sorbitol and trehalose are important sugars for thermotolerance in yeasts with their known ability to stabilize proteins, cell walls, and membranes [69], [35]. However, sorbitol did not suppress the ts phenotype of *ubp5*Δ, and the *ubp5*Δ mutant produced WT trehalose levels (data not shown). Therefore, the influence of Ubp5 on heat tolerance is likely independent of the trehalose pathway. Dozens of genes and several signaling pathways (such as MAPK, Ca^2+^/calcineurin, and Ras1 pathways) have also been identified as closely related to thermotolerance in *C. neoformans* [8], [70], [13]. For example, several mutants in the Ras1 pathway show a similar conditional ts phenotype, where the mutants are viable but fail to grow at 37°C or 39°C [71], [70], [72]. It remains to be proven whether Ubp5 is connected to the Ras1 pathway in regulation of thermotolerance in *C. neoformans*. Interestingly, no other deubiquitinating enzymes in *C. neoformans* appear to take part in regulation of thermotolerance other than Ubp5 [32]. In contrast, studies in *S. cerevisiae* reveal that several DUBs such as Doa4, Ubp8, Ubp10 and Ubp15 play roles in thermotolerance [22], and again illustrate both the functional similarities and divergences of DUBs between these two fungal species.

In addition to regulating thermotolerance, Ubp5 is also essential for the resistance of *C. neoformans* to other important biological stresses such as oxidative and nitrosative stress, salt stress, cell wall and cell membrane damaging agents, and antifungal agents (Figure 2 and Table 2). The ubiquitin-proteasome system has been identified as one of four core protein families in response to H_2_O_2_ stress across multiple eukaryotic species, including humans, plants, and both fission and budding yeasts [73]. A recent study demonstrated that the ubiquitin system is involved in both oxidative stress responses and in responses to antifungal agents such as fluconazole and amphotericin B in *C. neoformans* [33]. Consistent with these general phenotypic observations, our findings identified Ubp5 as specifically important for enhanced susceptibility to all four classes of antifungal agents, and suggest that a single deubquitinating enzyme may be implicated in the regulation of ergosterol synthesis, DNA/RNA biosynthesis and β-glucan formation in *C. neoformans,* which is also supported by our transcription profiling data (Tables 2 & 3). Along with Ubp5, we observed that the *doa4*Δ mutant also showed decreased resistance to high sodium ions, 0.02% SDS and 0.1% caffeine, and both *doa4*Δ and *ubp13*Δ mutants are more sensitive to fluconazole and caspofungin, demonstrating some functional overlap of the deubiquitinating enzymes in *C. neoformans*.

Presumably, various extracellular or intracellular stresses such as high temperature, oxidative and nitrosative damage, high salt, and antifungal drugs, lead to accumulation of misfolded or damaged proteins inside the yeast cell, and cause cell damage that is prevented by their removal through the ubiquitin-dependent proteolytic pathway. The importance of ubiquitin, as an essential element during stress responses, is further highlighted by the requirement of a functional polyubiquitin gene to withstand heat stress, elevated H_2_O_2_, and other stress agents in yeast [65], [74], [75]. Conversely, deubiquitination is not an essential pathway for general survival. The primary function of DUB enzymes is to maintain ubiquitin homeostasis via processing or recycling ubiquitin from its precursors or adducts [19]. Ubiquitin is likely encoded by two genes (*UBI4* and *UBI1*) in *C. neoformans*, which produce a linear peptide consisting of polyubiquitin (five copies) or monoubiquitin fused to a ribosomal protein [76]. Therefore, we hypothesize that Ubp5 in *C. neoformans* may be the major deubiquitinating enzyme to deal with stress. We suspect that it works synergistically with Ubi4 to maintain ubiquitin homeostasis especially under stressful conditions (Figure S3) and these specific regulatory connections will need to be further understood.

Several studies confirm that signaling pathways, such as PKC/MAPK, HOG, and Ca^2+^/ calcineurin pathways, jointly coordinate the stress responses in *C. neoformans* [4], [77], [13] and studies in other eukaryotic species reveal that the ubiquitin-proteasome system plays an important role in the regulation of these signaling pathways [78], [79], [80], [81]. For example, the ubiquitin system can down-regulate the HOG pathway via selective degradation of the upstream regulator Ssk1p in *S. cerevisiae* [78]. A recent report illustrates that the ubiquitin system may also play roles in osmotic stress, oxidative damage, and antifungal stress pathways by regulating the HOG pathway in *C. neoformans* [33]. Our microarray data also demonstrate that Ubp5 inactivation highly induces the transcription of several genes encoding MAPK and Ca^2+^/calcineurin signal pathway components such as *MAPKKK* and *CPB1*. Therefore, we hypothesize that there is complicated crosstalk between deubiquitinating enzymes and other signaling pathways in *C. neoformans* that require further study to understand their total impact on the pathobiology of *C. neoformans*.

In this study, we performed biological screening with both a macrophage-killing assay and a *G. mellonella* infection model. These models assess different aspects of cryptococcal pathogenesis and allow us to examine the virulence composite differences of each DUB mutant in a biological context (Figure 6 and Figure 7A?B). We found that deletions in either the *UBP5* or *DOA4* led to dramatic attenuation in virulence in both models and there were no significant virulence differences between *ubp5*Δ and *doa4*Δ mutants in either model, although disruption of *UBP5* did yield more altered in vitro phenotypes under several conditions. Reduced survival in the macrophage assay for both *ubp5*Δ and *doa4*Δ was observed, however, the reconstituted *ubp5*Δ*+UBP5* strain was unable to restore survival for the *ubp5*Δ mutant in this assay. This result may be due to the ectopic integration of *UBP5* in this strain suggesting that precise transcriptional control of *UBP5* is required; alternatively, a secondary mutation may exist in this strain.

Our results showing attenuated virulence of the *doa4*Δ mutant in *C. neoformans* was consistent with a prior report on this mutant in the mouse inhalation model [32]. It should be noted that the *doa4*Δ mutant was not complemented in this prior work, nor in this current report. It should also be pointed out that the *ubp14*Δ mutant (in strain H99) displays wild-type virulence in our assay (*P* = 0.995) while the same mutant in the gene-deletion library (in strain CMO18) showed decreased lung infectivity in the mouse model [32]. These contradictory results may be attributed to differences between vertebrate and non-vertebrate hosts, and/or the known variation of virulence between the different parental strains.

We also used the mouse inhalation model to test the virulence of the *ubp5*Δ mutant. All the mice infected by the *ubp5*Δ mutant survived for 8 weeks with low burden of yeasts in lung tissue and no viable yeast cells in CNS. With the long-term yeast survival in lung tissue of mice without dissemination and attenuation in the room temperature heterologous host model (*G. mellonella*), clearly, thermotolerance is not the only virulence factor influenced by Ubp5.

*C. neoformans* virulence has been linked to several other well-defined factors, including the production of capsule, melanin, and various lytic enzymes [82], [83], [84]. Of these classical virulence associated traits, capsule attachment was altered in the *ubp5*Δ mutant (Figure 3). Interestingly, a small proportion of mutant cells (10%) still exhibited normal capsule production although most showed an apparent capsular defect. Capsule production is a complex and dynamic process including intracellular polysaccharide synthesis, secretion of capsular polysaccharide via vesicles, and extracellular assembly [85]. Our capsule assay indicates that *UBP5* deletion does not affect capsule synthesis or secretion (Figure 3D), therefore Ubp5 probably participates in the polysaccharide assembly on the *C. neoformans* cell wall. Previous studies have identified that cell wall components such as alpha (1–3) glucan and chitin-derived molecules are required for GXM (major capsule component) anchoring to the cell surface [37], [86], [87]. In fact, enhanced susceptibility to cell wall-damaging agents such as caspofungin in the *ubp5*Δ mutant provides indirect evidence for its impact on cell wall formation. It has been reported that deletion of the transcriptional regulator *RIM101* produces a similar capsule defect by altering the expression of genes responsible for capsule attachment to the cell wall [88]. However, we examined the transcriptional expression of *AGS1* (encoding alpha-1,3-glucan synthase, key enzyme cell wall synthesis) and no significant differences were observed between *ubp5*Δ mutant and WT strain. It is possible that Ubp5 may be participating in regulation or modification of chitin-related structures or cell-wall components other than alpha-1,3-glucan for capsule assembly, which is also supported by our transcriptome profiling analysis in the *ubp5*△ mutant. On the other hand, ubiquitination and deubiquitnation are essential post-transcriptional modifications for many protein targets. Ubp5 may regulate the synthesis or degradation of the proteins (including Ags1) essential for capsule attachment at the protein translation level.

Our melanization assay did demonstrate some functional crosstalk among deubiquitinating enzymes in *C. neoformans*. In our study, several deubiquitinating enzymes (such as Ubp5, Doa4, Ubp13 and Ubp14) influenced pigment production in *C. neoformans* by their common coordination of intracellular copper homeostasis (Figure 4). Disruption of these genes resulted in hypomelanized colonies of *C. neoformans* at 37°C, a phenotype that can be suppressed by additional exogenous copper ions. Similar results were reported with two other genes, *CCC2* (copper transporter) and *ATX1* (copper chaperone), both of which could mediate intracellular transport of copper ion to regulate laccase transcription [39] . The melanin defect was not as profound in the other DUB mutants at 37°C, and high temperature may disturb regulation of copper metabolism. However, melanization is impacted with the *ubp5*Δ mutant at 30°C and this may be related to its decreased growth rate. Our results with H99 stand in contrast to a previous study [32], where deletion of *DOA4* or *UBP14* led to a melanin defect in the CMO18 strain at 30°C. Melanization generally provides *C. neoformans* an added survival advantage against both environmental predators and the mammalian host immune system [89], [90], [7]. However, disruption of either *UBP13* or *UBP14* does not attenuate the virulence of *C. neoformans* in both macrophage and wax moth killing assays as the *ubp5*Δ and *doa4*Δ mutants do despite their effects on melanization. This finding suggests that hypomelanization may not be a critical factor for attenuated virulence in the *ubp5*Δ and *doa4*Δ mutants and specifically, Doa4 may depend on other unrecognized mechanisms to regulate pathogenesis in *C. neoformans*.

In summary, we have clearly demonstrated that deubiquitinating enzymes, especially Ubp5, are necessary for the efficient phenotypic expression of multiple virulence factors in the model fungal pathogen *C. neoformans*. These enzymes play pivotal roles in regulating propagation, sporulation, and stress responses. Their distinct roles further illustrate the evolutionary divergence of certain pathogenic fungi from the model yeasts such as *S. cerevisiae*, and also provide an important paradigm for further exploring the functions and mechanisms of deubiquitinating enzymes on the virulence of other fungal pathogens, such as *Candida albicans*, *A. fumigatus*, *Ustilago maydis*, and *Magnaporthe grisea*. Furthermore, the pleiotropic phenotypes of Ubp5 highlight its potential as an antifungal drug target. Indeed, inhibitors of some deubiquitinating enzymes including Usp7 (Ubp5’s homolog in human) are current viable targets for anticancer therapy [91]. Therefore, we propose that specific deubiquitinating enzyme inhibitors could provide similar benefits in the prophylaxis and treatment of fungal diseases.

## Materials and Methods

### Strains, Plasmids, and Media

The *C. neoformans* strains used in this study are listed in Table 1. Strains were cultured at 30°C on YPD agar medium, and selective media contained nourseothricin (100 mg L^−1^) and/or neomycin (G418 200 mg L^−1^). Capsule-inducing medium, urease test medium and DOPA were prepared as previously reported [92], [93]. Stress media were created by adding different stress-inducing agents into YNB agar medium (0.67% yeast nitrogen base without amino acids, 2% glucose) or YPD agar medium before autoclaving. V8 medium (pH = 5.0) was used for mating and cell infusion assays. Medium for J774 murine macrophages was prepared as previously reported [94].

### Transcriptional Profiling

Transcriptional profiles were generated for WT and previously described mutant strains [95] from either ex vivo CSF and serum cultures or in vivo rabbit and human samples. In the comparison of CSF and serum exposure, WT strain H99 was incubated in two 50 mL YPD broth cultures for 24 hours at 30°C with shaking, washed with sterile distilled water, and resuspended in 50 mL sterile distilled water. The cultures were then split in half and pelleted by centrifugation, with half of the cultures resuspended in filter-sterilized human CSF or serum from anonymous human donors. These cultures were then incubated at 37°C with shaking, and harvested at either 1 or 24 hours post-resuspension in each biological fluid for RNA extraction. In the comparison of ex vivo CSF and rabbit infection, WT strain H99 was grown as described above, resuspended in ex vivo CSF for 24 hours, and harvested for RNA extraction. In the comparison of rabbit and human samples, New Zealand White rabbits were inoculated with WT strain H99 and harvested by intracisternal spinal tap on days 1 and 7 as previously described [95]. For the human sample, RNA was extracted from CSF obtained from a de-identified patient with cryptococcal meningitis as previously described [96]. RNA for all samples was obtained from lyophilized cells using the TriZol extraction protocol (Invitrogen, Carlsbad, CA) followed by purification using the Qiagen RNeasy kit (Qiagen, Valencia, CA) as previously described [96].

In the comparison between *ubp5*△ mutant and wild-type strain, both strains were inoculated in 50 mL YPD medium and grown to mid-log phase (OD_600_?1.5) at 30°C. The cultures were then split into two parts and pelleted by centrifugation. One part of the cultures resuspended in 25 mL fresh YPD medium and incubated for 1 h at 37°C. Following RNA extraction, microarray hybridizations and data acquisition were performed at the Duke Microarray Facility (http://microarray.genome.duke.edu/) according to their established protocols for custom spotted arrays. All transcription profiling data generated for this study have been deposited in the Gene Expression Omnibus, a MIAME-compliant database at the National Center for Biotechnology Information, under Accession # GSE35067.

### Construction of DUB Mutant Strains and Reconstitution

Each DUB gene was disrupted in H99 and/or KN99a strains background by overlap PCR [97]. The purified PCR fragments were reduced to a 2 µl volume and precipitated onto 10 µl gold microparticles (0.6 µm; Bio-Rad) for biolistic transformation [98]. Stable transformants were selected by plating cells onto YPD medium containing either neomycin or nourseothricin. Mutants were screened by PCR using appropriate primers (Table S1). Positive transformants identified by PCR screening were further confirmed by Southern blot analysis.

To complement the *ubp5*Δ mutant, a genomic DNA fragment containing ORF, promoter and terminator region was amplified using primers Re0104-F and Re0104-R. This PCR fragment and the digested plasmid pCH233 by Xba I were fused with In-Fusion® EcoDry™ Cloning System (Clontech). The reconstructed vector pUBP5-NAT was reintroduced into *ubp5*Δ mutants via biolistic transformation. Positive colonies were selected on YPD agar plus nourseothricin. Reconstitution was confirmed by PCR and Southern hybridization.

### Southern Blot Analysis

The mutant and reconstituted strains were reconfirmed using Southern hybridization analysis for gene replacement. Genomic DNA (20 µg) was digested with proper restriction endonucleases and separated on a 0.8% agarose gel. The DNA was transferred to a positively charged nylon membrane (Roche Applied Science, Indianapolis, IN). Membranes were hybridized with NEO, NAT or target-gene digoxigenin-labeled DNA probes overnight, as specified by the manufacturer (Roche Applied Science, Indianapolis, IN) washed and detected on the membrane [94]. DNA bands of interest were visualized on film following 3 to 10 min exposures.

### RNA Extraction, cDNA Synthesis and Quantitative Real-time PCR

Following incubation to mid-log phase, 50 ml of *C. neoformans* cells were harvested by centrifugation at 1,800 ×*g* for 5 min, washed once with 1× PBS, and lyophilized overnight. The lyophilized pellet was then vortexed with 3 ml glass beads and resuspended in 4 ml TRIzol Reagent (Sigma). After sitting at room temperature for 5 min, 800 ml chloroform was added and the mixture was shaken for 30 s. The cell lysate was then centrifuged at 8,000 ×*g* for 10 min, and the supernatant was transferred to a new tube. Same volume of isopropanol was added, incubated for 10 min at room temperature, and centrifuged at 8,000 ×*g* for 10 min. After washing the pellet with 75% ethanol, it was diluted into 300 µl RNase-free water and incubated with DNase I at 37°C for 1 h. The RNA was purified with RNeasy Mini Kit (QIAGEN) again, and then stored at −80°C. First-strand cDNA was synthesized using random hexamer primers via the SuperScript III First-Strand Synthesis Kit for RT-PCR (Invitrogen) according to its protocol.

The resulting cDNA was used as template for quantitative real-time PCR using iQ SYBR Green Supermix (Bio-Rad) according to the manufacturer’s recommendations. Primers used for each gene are listed in Table S1 in the supplemental material. The multicolor real-time detection system was used with the following PCR conditions: an initial denaturing cycle of 95°C for 3 min and 40 cycles of denaturation at 95°C for 10 s and annealing/extension at 55°C for 20 s. Each reaction was performed in triplicate. The constitutively expressed GPD1 gene was used to normalize gene amplification for each strain and condition [99], and expression (n-fold) was calculated relative to the WT strain H99 using the Bio-Rad iCycler software system as previously described [93].

### In vitro Stress Assays

Each strain was incubated to saturation at 30°C in YPD medium, washed, serially diluted (1 to 10^6^dilutions) in 1× PBS, and spotted (3 µl) onto YNB or YPD agar medium containing different stress-inducing agents. For the oxidative and NO stress test, 2 mM H_2_O_2_ and 0.75 mM NaNO_2_ (pH = 4.0) were added into YNB agar medium. For the osmotic stress and high salt sensitivity test, we added 1.5 M sorbitol, 1.5 M NaCl, 1.5 M KCl to YPD agar medium. To examine cell wall integrity, the cells were spotted onto YPD agar media containing 0.02% SDS, 0.1% caffeine and 0.5% congo red. Then spotted cells were incubated at 30°C for 5 days and photographed.

All the mutant strains, along with WT, were analyzed for their sensitivities to the stress of antifungal compounds, such as amphotericin B, fluconazole, flucytosine and caspofungin. Tests were performed in accordance with NCCLS protocol M-27A [35], and *Candida parapsilosis* ATCC22019 was used as a quality control strain. The MIC_80_ was determined to compare the antifungal activity among different strains.

### Assays for Melanization, Urease, Capsule Production

For the melanization test, cells were spotted onto L-DOPA agar and incubated for 3–7 days at 30°C and 37°C. Melanin production was monitored and photographed. For urease assay, strains were streaked onto Christiansen’s urea agar at 30°C for 24 hours and checked for the color change with WT and *ure1*Δ strains as controls [92].

For capsule production, different *C. neoformans* strains were incubated into DME medium or low iron medium at 37°C in 5% CO_2_ for 3 days in tissue culture flasks. Following capsule induction, the cells were washed once with 1× PBS, and resuspended in 1 ml sterile PBS for visualization of capsule. Each strain was mixed with India ink preparations on glass slides for visualization. Images were collected at a 100× magnification. Total (cell and capsule) and cell-only diameters were compared for 50 cells for each strain. Cryptocrit was also used to measure differences in capsule [93]. In order to examine the capsule secretion, a capsule transfer assay was performed per previous studies [37]. Briefly, conditioned medium (CM) was prepared as a source of capsule polysaccharide by growing WT and *ubp5*Δ mutant cells in DMEM medium for one week while CM from acapsular cells (*cap59*Δ) as a negative control, after which 2.5×10^6^ of acapsular acceptor cells (*cap59*Δ) were incubated in the CM media. For indirect immunofluorescence, cells were co-cultured for one hour with monoclonal antibody mAb18B7 and then combined with secondary antibody (Alexa Fluror anti-mouse IgG 2 mg ml^−1^, Invitrogen). Cells were visualized on a Zeiss LSM inverted confocal microscope with the Argon/2 488 laser at 100×magnification.

### Mating and Cell Fusion Assays

In a mating assay, strains of opposite mating type were mixed and co-cultured on V8 agar medium at 25°C in the dark. Mating filaments and basidiospore formation were examined and recorded by photography using a Zeiss Axio Imager A1 fluorescent microscope equipped with an AxioCam MRM digital camera after two weeks. Also, a cell fusion assay was performed [100]. 10^7^ cells ml^−1^ of each mutant containing Nat^R^ or Neo^R^ markers (DF001 and DF002) were mixed together in an equal volume and 5 µl of the mixture was spotted onto V8 medium and incubated for 24 h at 25°C in the dark. Scraped cells from the V8 medium, were resuspended in 1 ml dH_2_O, and then 200 µl of cell suspension were spread onto YPD medium containing nourseothricin and neomycin. The numbers of colonies on each plate were counted after 4 days of incubation at room temperature. TOC4 (Neo^r^ wild-type strain) and DF010 (Nat^r^ wild-type strain) were used as control strains.

### Macrophage Killing Assays

We assessed *ubp5*Δ mutant and its complemented strains for survival within macrophages. Yeast strains were incubated overnight at 30°C. J774A.1 macrophages at a concentration of 10^6^ per well were activated by gamma interferon-γ and lipopolysaccharide, then inoculated with yeasts for the macrophage-yeast survival assay [94]. Briefly, activated macrophages were coincubated with 10^6^ yeast cells of each strain for 2 h at 37°C in 5% CO_2_ to allow phagocytosis. Prior to co-incubation, cells of each yeast strain were incubated with the monoclonal antibody mAb18B7 for one hour. Then extracellular yeasts were washed away three times with 1×PBS and the monolayers were incubated in DMEM. For the macrophage-yeast killing assay, washed monolayer cells containing yeast and macrophage cells were co-incubated overnight (24 hr). Macrophages were then lysed with 0.5% SDS, and lysates were diluted and plated on YPD to count numbers of viable yeasts present following incubation at 30°C for 3 days.

### Galleria Mellonella Virulence Assay

*G. mellonella* caterpillars (330±25 mg in body weight) in the final instar larval stage were employed in our assays. Sixteen caterpillars of the required weight were randomly chosen in each group. A 10 ul Hamilton syringe was used to inject 10 ul aliquots of the yeast inoculum (5×10^5^ cells) into the hemocoel of each caterpillar via the last left proleg as described previously [52], and these caterpillars were incubated in plastic containers at room temperature. Caterpillars were considered dead when they displayed no movement in response to touch. The number of dead caterpillars was recorded daily. Each assay was performed in duplicate.

### Murine Virulence Assay

Virulence of the *ubp5*Δ mutant strain was assessed in the inhalational mouse model according to an established protocol [9]. A/Jcr mice (10 mice for each *C. neoformans* strain) were infected intranasally with 10^5^ CFU of either WT (H99), mutant (*ubp5*Δ), or reconstituted (*ubp5*Δ+ *UBP5*) strains of *C. neoformans* in a volume of 50 µl sterile PBS. The mice were monitored closely for signs of worsening infection and euthanized at predetermined endpoints (weight loss of ≥15%, neurological symptoms, and an inability to access food/water).

### Statistics

Data from the survival assays in both *G. mellonella* and murine infection models were plotted using Kaplan-Meier survival curves and analyzed by the log rank test (SPSS 15.0). All the other statistical data were calculated and compared using (Student *t* tests). *P*<0.05 were considered statistically significant.

### Ethics Statement

This study was carried out in strict accordance with the recommendations in the Guide for the Care and Use of Laboratory Animals of the National Institutes of Health. The protocol was approved by the Institutional Animal Care and Use Committee of Duke University (Protocol Number: A266-08-10). Infections were carried out under isofluorane anesthesia, and all efforts were made to minimize suffering.

## Supporting Information

Figure S1 **Growth rate measurement for the** ***ubp5***Δ **mutant.** WT, *ubp5*Δ and *ubp5*Δ*+UBP5* strains were grown overnight at 30°C in liquid YPD medium. Cell numbers were counted by hemacytometer. 10^6^ CFU from each culture were transferred to 30 ml fresh YPD medium in flasks and incubated at 30°C. OD_600_ was measured for each culture at four hour intervals. Mutant *ubp5*Δ showed slower growth than H99 at 30°C.
(TIF)

Figure S2 **Urease assay.** Different DUB mutants grew on Christensen’s medium at 30°C for the detection of urease production. *ure1Δ* mutant strain was used as a negative control.
(TIF)

Figure S3 ***UBP5*** **deletion lead to increased expression of** ***UBI4*** **in** ***C. neoformans*** **.** Expression of polyubiquitin gene *UBI4* at (A) 30°C and (B) 37°C. *UBI4* was up-regulated in *ubp5*Δ mutant while the reconstituted strain showed similar expression level like WT strain. And high temperature exposure reinforced the up-regulation of *UBI4* expression in *ubp5*Δ strain.
(TIF)

Table S1 **Primers used in this study.** Primers were designed using the Primer3 software package (http://frodo.wi.mit.edu/primer3/) for generating gene-deletion cassettes, gene-reconstitution cassettes, Southern hybridization probes, or real-time PCR products.
(PDF)

Table S2 **Ubiquitin-proteasome genes identified by in vivo transcription profiling.** Transcription profiles of in vivo *C. neoformans* cells from human or rabbit infections was compared to profiles from *C. neoformans* cells grown ex vivo in either pooled human cerebrospinal fluid or serum to identify genes that are differentially expressed in the host.
(PDF)
