# Supplementary figures and images for: Pleiotropic Effects of Deubiquitinating Enzyme Ubp5 on Growth and Pathogenesis of Cryptococcus neoformans
Source: PLoS One. 2012 Jun 14;7(6):e38326. doi: 10.1371/journal.pone.0038326 (PMC3375289; doi:10.1371/journal.pone.0038326)

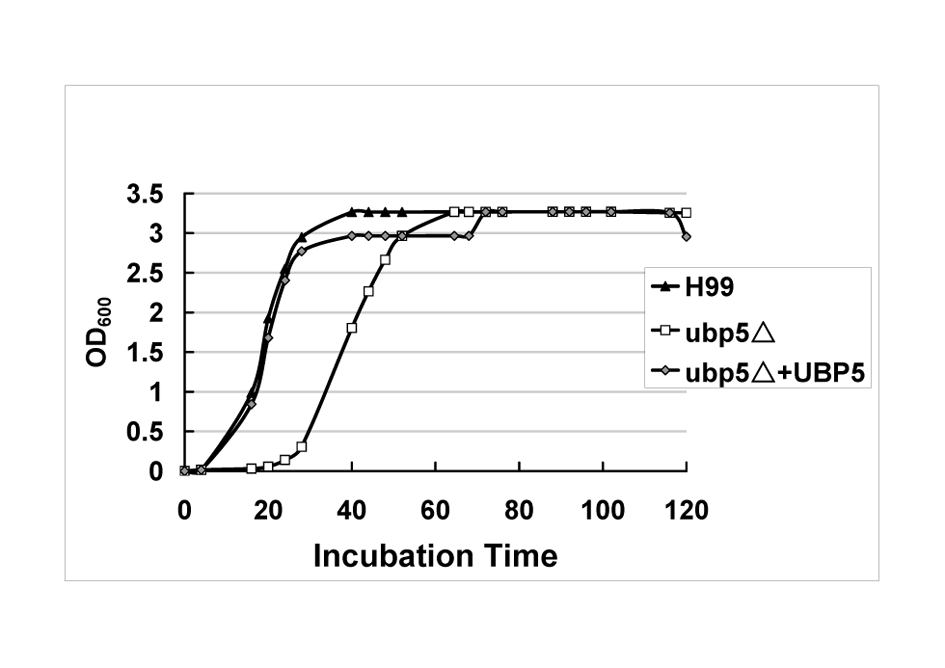

Supplement: Figure S1 — Growth rate measurement for the ubp5Δ mutant. WT, ubp5Δ and ubp5Δ+UBP5 strains were grown overnight at 30°C in liquid YPD medium. Cell numbers were counted by hemacytometer. 106 CFU from each culture were transferred to 30 ml fresh YPD medium in flasks and incubated at 30°C. OD600 was measured for each culture at four hour intervals. Mutant ubp5Δ showed slower growth than H99 at 30°C. (TIF) [file pone.0038326.s001.tif]

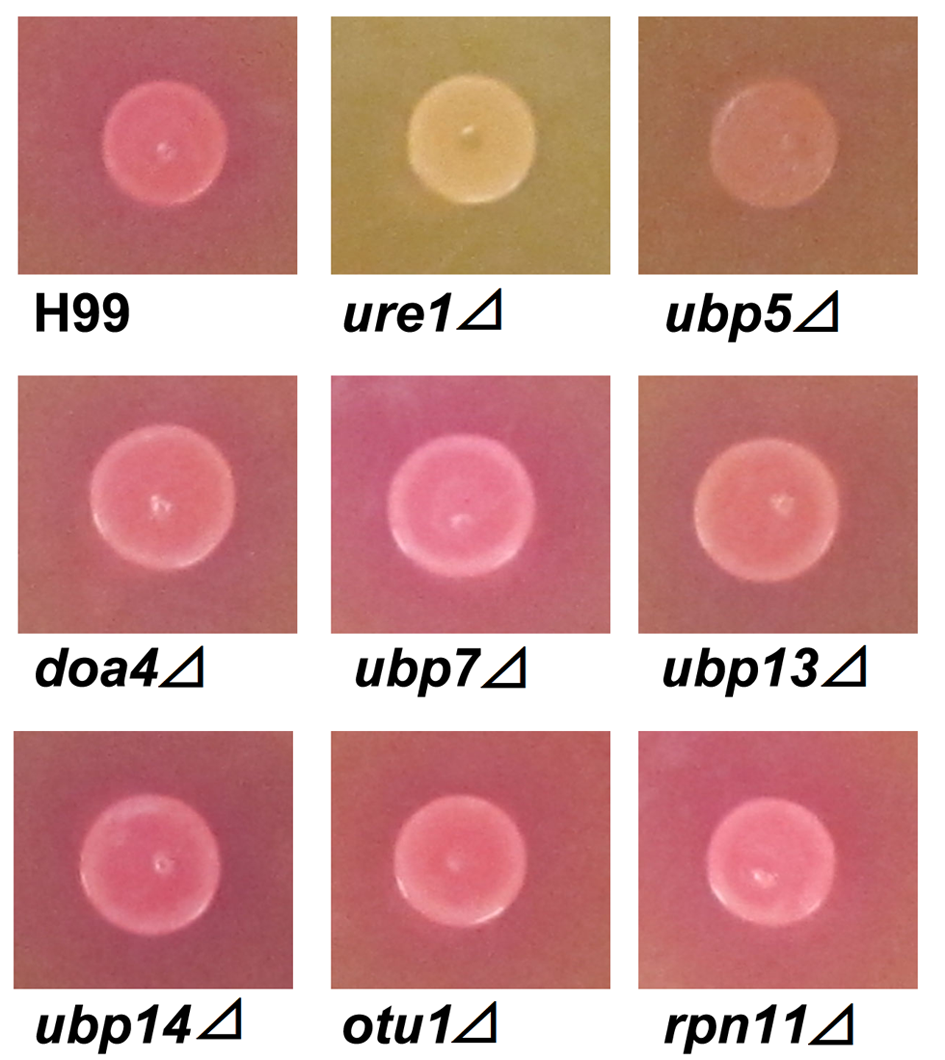

Supplement: Figure S2 — Urease assay. Different DUB mutants grew on Christensen’s medium at 30°C for the detection of urease production. ure1Δ mutant strain was used as a negative control. (TIF) [file pone.0038326.s002.tif]

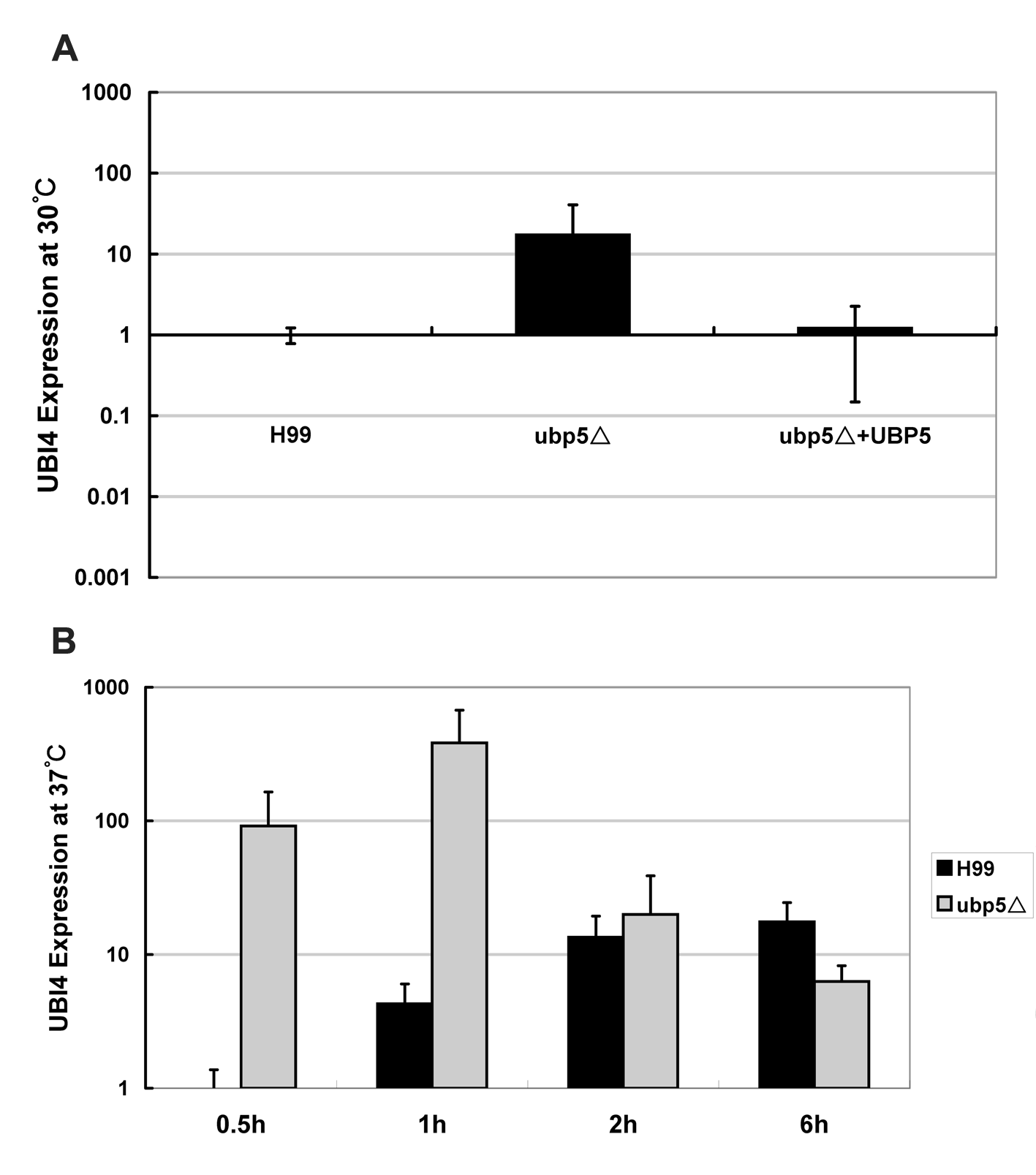

Supplement: Figure S3 — UBP5 deletion lead to increased expression of UBI4 in C. neoformans . Expression of polyubiquitin gene UBI4 at (A) 30°C and (B) 37°C. UBI4 was up-regulated in ubp5Δ mutant while the reconstituted strain showed similar expression level like WT strain. And high temperature exposure reinforced the up-regulation of UBI4 expression in ubp5Δ strain. (TIF) [file pone.0038326.s003.tif]
