# Supplementary material for: Pleiotropic Effects of Deubiquitinating Enzyme Ubp5 on Growth and Pathogenesis of Cryptococcus neoformans
Source: PLoS One. 2012 Jun 14;7(6):e38326. doi: 10.1371/journal.pone.0038326 (PMC3375289; doi:10.1371/journal.pone.0038326)
Supplement: Table S1 — Primers used in this study. Primers were designed using the Primer3 software package (http://frodo.wi.mit.edu/primer3/) for generating gene-deletion cassettes, gene-reconstitution cassettes, Southern hybridization probes, or real-time PCR products. (PDF) [file pone.0038326.s004.pdf]

Table S1. Primers used in this study.

Primer	Sequence(5'-3')	Function
WF0001	CTGCCAAGAGTAAAGCAGAT	Constructing of knocking-out cassatte/UBP5
WF0002	GTCATAGCTGTTTCCTGGTCAACATCTCGTCCAAACT	Constructing of knocking-out cassatte/UBP5
WF0003	CTGGCCGTCGTTTTACTTACACACATTTGCGAACTC	Constructing of knocking-out cassatte
WF0004	GCAAGGTGCATAGACTAACC	Constructing of knocking-out cassatte/UBP5
WF0009	TGTCCTCTCAGCAACTTCTT	Constructing of knocking-out cassatte/OTU1
WF0010	GTCATAGCTGTTTCCTGCATCTATGGTGGTGGTTTCT	Constructing of knocking-out cassatte/OTU1
WF0011	CTGGCCGTCGTTTTACACCCATAATCCGAAGAGAAT	Constructing of knocking-out cassatte/OTU1
WF0012	CTTGGCTAACAAGTTCGTTT	Constructing of knocking-out cassatte/OTU1
WF0033	CTCCTTCTCTCGCTGCATCT	Constructing of knocking-out cassatte/RPN11
WF0034	GTCATAGCTGTTTCCTGACGCGAGCGTCATCTTCTAT	Constructing of knocking-out cassatte/RPN11
WF0035	CTGGCCGTCGTTTTACGCTCGAAAACGACAATGACA	Constructing of knocking-out cassatte/RPN11
WF0036	ACGTGGGATGCCTAGGTAAA	Constructing of knocking-out cassatte/RPN11
WF0037	TGCTCGGTAAGTCCTCAACC	Constructing of knocking-out cassatte/UBP13
WF0038	GTCATAGCTGTTTCCTGCCAAAGACAGGCGAGTAACC	Constructing of knocking-out cassatte/UBP13
WF0039	CTGGCCGTCGTTTTACGATTGTCAACCCCCATCATC	Constructing of knocking-out cassatte/UBP13
WF0040	TTGAGAGGACGTCTGTGGTG	Constructing of knocking-out cassatte/UBP13
WF0045	GATGGCCCCTTACTCATCCT	Constructing of knocking-out cassatte/UBP7
WF0046	GTCATAGCTGTTTCCTGCACAAGGAATGCCCTGAAAT	Constructing of knocking-out cassatte/UBP7
WF0047	CTGGCCGTCGTTTTACCACTTGACGCGTGTTGTTTT	Constructing of knocking-out cassatte/UBP7

WF0048	GGCAGAAGCTGTCCTCAAAC	Constructing of knocking-out cassatte/UBP7
WF0065	TTGGTCTATCCAGACACACA	Constructing of knocking-out cassatte/UBP14
WF0068	TATGGTCGTGATGAAGATGA	Constructing of knocking-out cassatte/UBP14
WF0069	GAGATTGGAGAGGATGTCAA	Constructing of knocking-out cassatte/DOA4
WF0070	GTCATAGCTGTTTCCTG GCGTTCGAACCATACTTTG	Constructing of knocking-out cassatte/DOA4
WF0071	CTGGCCGTCGTTTTAC AAGAAGAGTACTGCGATTGG	Constructing of knocking-out cassatte/DOA4
WF0072	AGGACAGAAGACAGGAAAAA	Constructing of knocking-out cassatte/DOA4
M13F	GTAAAACGACGGCCAG	Constructing of knocking-out cassatte
M13R	CAGGAAACAGCTATGAC	Constructing of knocking-out cassatte
WF0104-cp	TCCCAGCATTGTGATTGAAA	Analysis of proper homologous recombination/UBP5
WF0912-cp	GGTCACCCTTTTCATGCACT	Analysis of proper homologous recombination/OTU1
WF3336-cp	TCTGGCAAGTCAATGCTGTC	Analysis of proper homologous recombination/RPN11
WF3740-cp	TCGTCGTGGGTGATGTTAAA	Analysis of proper homologous recombination/UBP13
WF4548-cp	GAATCATTCCCCTCTCGTCA	Analysis of proper homologous recombination/UBP7
WF6568-cp	GCAGGGTGTATTATCGACAT	Analysis of proper homologous recombination/UBP14
WF6972-cp	CAAACATAACATGACGCATC	Analysis of proper homologous recombination/DOA4
Re0104-F	CGAGCATGCATCTAGAACTCAAAACGAGCAGAACAT	Reconstitiution for Cn-*ubp5*Δ mutant
Re0104-R	AATTGGGCCCTCTAGAAGCTTTAGACTGCAAGAGC	Reconstitiution for Cn-*ubp5*Δ mutant
Probe14-F	CGAATTGAGCACTGGAGTCA	Creation of UBP5 Probe for Soutern analysis
Probe14-R	AGTACGGGCCCTTCTTTGTT	Creation of UBP5 Probe for Soutern analysis

Neo-F	TATGTCCTGATAGCGGTCCG	Creation of NEO Probe for Soutern analysis
Neo-R	AAGATGGATTGCACGCAGG	Creation of NEO Probe for Soutern analysis
Nat-F	ACCTCTGGCTGGAGGTCAC	Creation of NAT Probe for Soutern analysis
Nat-R	GGGCATGCTCATGTAGAGC	Creation of NAT Probe for Soutern analysis
GPD1-a	AGTATGACTCCAACAATGGTCG	Real time PCR
GPD1-b	AGACAAACATCGGAGCATCAGC	Real time PCR
AGS1-a	TACCGATGGCCCTTTTACTCTA	Real time PCR
AGS1-b	AGGAATACACCATGGATGGAAG	Real time PCR
UBI4-a	CACCTTGTTCTCCGTCTG	Real time PCR
UBI4-b	TTGGAGGATGAGGACAATC	Real time PCR
MFα-a	ACGCCTTCACTGCCATCTTC	Real time PCR
MFα-b	GACGCATAGGGTCATGCCAC	Real time PCR
