# Supplementary material for: Pleiotropic Effects of Deubiquitinating Enzyme Ubp5 on Growth and Pathogenesis of Cryptococcus neoformans
Source: PLoS One. 2012 Jun 14;7(6):e38326. doi: 10.1371/journal.pone.0038326 (PMC3375289; doi:10.1371/journal.pone.0038326)
Supplement: Table S2 — Ubiquitin-proteasome genes identified by in vivo transcription profiling. Transcription profiles of in vivo C. neoformans cells from human or rabbit infections was compared to profiles from C. neoformans cells grown ex vivo in either pooled human cerebrospinal fluid or serum to identify genes that are differentially expressed in the host. (PDF) [file pone.0038326.s005.pdf]

Table S2. Ubiquitin-proteasome genes identified by in vivo transcription profiling.

ID[a]	Gene Name	Annotation
CNA02100	*RPN11*	DUBs, JAMM family
CNA03330	*UBI1*	Ubiquitin precursor
CNA07360	*DOA4*	DUBs, USP family
CNAG_05098	*HUB1*	UB-like molecule
CNB03140	*Hrt1*	Rub1 ligation enzyme
CNC01280	*UBP10*	DUBs, USP family
CNC02000	*UBA3*	Rub1 activating enzyme
CNC04440	*RUB1*	UB-like molecule
CND01140	*UBA4*	Urm1 activating enzyme
CND01170	*UCH*	DUBs, UCH family
CND05210	*DCN1*	Rub1 ligation enzyme
CNH02890	*UBP5*	DUBs, USP family
CNJ01020	*UBC12*	Rub1 conjugation enzyme
CNK02620	*UBI4*	Ubiquitin precursor
CNL05240	*HUB1*	UB-like molecule
CNL05330	*URM1*	UB-like molecule

[a]Broad or NCBI Locus ID
